# Genome wide study of cysteine rich receptor like proteins in *Gossypium* sp.

**DOI:** 10.1038/s41598-022-08943-1

**Published:** 2022-03-22

**Authors:** Athar Hussain, Naila Asif, Abdul Rafay Pirzada, Azka Noureen, Javeria Shaukat, Akif Burhan, Madiha Zaynab, Ejaz Ali, Koukab Imran, Ayesha Ameen, Muhammad Arslan Mahmood, Aquib Nazar, M. Shahid Mukhtar

**Affiliations:** 1 Genomics Lab, School of Food and Agricultural Sciences (SFAS), University of Management and Technology (UMT), Lahore, 54000 Pakistan; 2 National Institute for Biotechnology and Genetic Engineering (NIBGE), College of Pakistan Institute of Engineering and Applied Sciences (PIEAS), Faisalabad, 38000 Pakistan; 3 Department of Life Sciences, School of Science, University of Management and Technology (UMT), Lahore, 54000 Pakistan; 4 PMAS-Arid Agriculture University Rawalpindi, Rawalpindi, 46300 Pakistan; 5 Shenzhen Key Laboratory of Marine Bioresource & Eco-Environmental Sciences, College of Life Sciences and Oceanography, Shenzhen University, Shenzhen, 51807 China; 6 Center of Excellence in Molecular Biology, University of Punjab, Lahore, 54000 Pakistan; 7 Office of Research Innovation and Commercialization, University of Management and Technology (UMT), Lahore, 54000 Pakistan; 8 Department of Biology, the University of Alabama at Birmingham, 1300 University Blvd., Birmingham, AL 35294 USA

**Keywords:** Agricultural genetics, Plant evolution, Abiotic, Biotic

## Abstract

Cysteine-rich receptor-like-kinases (*CRKs*), a transmembrane subfamily of receptor-like kinase, play crucial roles in plant adaptation. As such cotton is the major source of fiber for the textile industry, but environmental stresses are limiting its growth and production. Here, we have performed a deep computational analysis of *CRKs* in five *Gossypium* species, including *G. arboreum* (60 genes)*, G. raimondii* (74 genes)*, G. herbaceum* (65 genes)*, G. hirsutum* (118 genes), and *G. barbadense* (120 genes). All identified CRKs were classified into 11 major classes and 43 subclasses with the finding of several novel CRK-associated domains including *ALMT, FUSC_2, Cript, FYVE,* and *Pkinase*. Of these, *DUF26_DUF26_Pkinase_Tyr* was common and had elevated expression under different biotic and abiotic stresses. Moreover, the 35 land plants comparison identified several new *CRKs* domain-architectures. Likewise, several SNPs and InDels were observed in CLCuD resistant *G. hirsutum*. The miRNA target side prediction and their expression profiling in different tissues predicted *miR172* as a major CRK regulating miR. The expression profiling of *CRKs* identified multiple clusters with co-expression under certain stress conditions. The expression analysis under CLCuD highlighted the role of *GhCRK057, GhCRK059, GhCRK058, and GhCRK081* in resistant accession. Overall, these results provided primary data for future potential functional analysis as well as a reference study for other agronomically important crops.

## Introduction

In nature, plants are exposed to diverse environmental stresses, including biotic and abiotic stresses. To defend against these stresses, plants have evolved layered immune systems. This includes patterns-triggered immunity (PTI)^1^ that is induced by pattern recognition receptors (PRRs)^2^. Whereas effector-triggered immune (ETI) is activated when plants detect pathogens' RNAs and proteins-based effector molecules. From pathogens’ perspective, these effectors suppress both PTI and ETI and establish effector-triggered susceptibility (ETS)^3–5^. The plasma membrane possesses embedded proteins with extracellular and intracellular domains, including receptor-like kinases (RLKs) and receptor-like proteins (RLPs)^6^ that generally participate in PTI. The extracellular domain involves host–pathogen protein–protein interaction and signal perception, while the intracellular kinase domains transduce that signal and activate signaling pathways^7–12^. The RLKs have a potential role in different signaling mechanisms, including stress responses, hormone regulation, and plant growth and development^13,14^. RLKs and RLPs involve in regulations of several cellular mechanisms to strengthen plant adaptation under different environmental stresses. Multiple genome-wide studies in plants have identified RLKs and RLPs, but fewer have been biochemically and functionally characterized. The evolutionary divergence and speciation have been triggered for subfunctionalization and neofunctionalization of proteins, including RLKs and RLPs.

Cysteine-rich receptor-like kinase (*CRKs*), harboring Domain Unknown Function 26 (DUF 26; Gnk2 or Stress-antifungal) domain, is an extracellular domain that consists of the conserved cysteine-rich motif (C-X8-C-X2-C) in its core and possesses antifungal and salt-stress responsive activities. Thus far, the best-characterized *CRKs* identified in *Gingko biloba* consists of a single DUF26, which acts as mannose-binding lectin and provides resistance against the fungal pathogen^15^. The structural analysis of Arabidopsis PDLP5 and PDLP8 ectodomains is also similar to fungal lectins but in plants, it has an additional domain for carbohydrate-binding^16^. In Arabidopsis, the AtCRKs are transcriptionally induced under abiotic stresses such as salt, drought, UV light, heat, salicylic acid^17–21^. In addition, a subset of *CRKs* is strongly induced in response to pathogens and pathogen-mimic stimuli^19,20^. Similarly, the overexpression of Arabidopsis *CRK4*, *CRK5*, *CRK6*, *CRK13*, and *CRK36* exhibited enhanced resistance to a bacterial pathogen *Pseudomonas syringae* and activated both early and late PTI responses^17,21,22^. The *CRKs* are categorized into three subgroups including cysteine-rich receptor-like secreted proteins (CRRSPs; single peptide followed by DUF26), cysteine-rich receptor-like protein kinases (*CRKs*; single peptide, two DUF26 domains, one transmembrane domain, and one kinase domain), plasmodesmata localized proteins (PDPs; with single DUF26 domain). These are involved in pathogen response, intra signaling, systematic signaling, and viral movement target^23^. A recent study identified CRKs in 32 plant species and algae genomes and classified them into nine subclasses i.e., sdCRRSP, ddCRRSP, PDLP, sdCRK, CRK_I, CRK_II, tdCRK, qdCRK, and qdCRRSP^15^. While the essential roles in plant adaptation are documented, their functions in *Gossypium* sp. are not explored.

The *Gossypium* genus encompasses 54 species with 47 diploids (2n = 26), and seven tetraploids (2n = 4x = 52)^24^. Among these species, only four are widely cultivated globally for fiber production. This includes two tetraploids (*G. hirsutum*; A_t_D_t_1 and *G. barbadense*; A_t_D_t_2) and two diploids (*G. arboreum*; A2, *G. herbaceum*; A1) species. According to the cotton polyploidization theory, the tetraploid AADD genome originated due to polyploidization of the A-like genome and D-like genome^25–30^. It is reported that the diploid species are resistant to several viral and fungal diseases as compared to allotetraploid^28,31,32^. Thus, a comparative study among resistant and susceptible species are essential to understand plant resistance mechanism for developing resistant cultivars. The current study comprises of genomic, transcriptomic, proteomic, and miRNA target site prediction study of CRK genes among five species, including *G. arboreum, G. raimondii, G. herbaceum, G. barbadense,* and *G. hirsutum.* Findings of this study have provided comprehensive insight into the *CRKs*’ evolution, expression patterns, interaction with viral proteins, genetic diversity of resistant and susceptible accessions, and miRNA target site predictions in *Gossypium* sp.

## Material and methods

### Identification and classification

The complete genome of *G. hirsutum* (*Ghir: HAU_v1/v1.1*)*, G. barbadense (Gbar; genome HAU_v2_a1*), *G. herbaceum (Gher; A1-0076_WHUv3.0), G. raimondii (Gra; BGI-CGP_v1.0), and G. arboreum (Gar; CRI-A2_v1.0_a1.0*) and their associated data were retrieved from Cottongen and CottonFGD databases^25,27,28,33–35^. These protein sequences were scanned through the *Pfam* database in the local server using the *Pfam-Scan* tool^36^ with default parameters. All genes having *DUF 26* (PF01657; Gnk2 or Stress-antifungal) domains were considered as *CRKs*. The identified proteins sequence was scanned with the Inter-Pro database and filtered with IPR038408 and IPR002902 accessions for further validation. In addition, we have also mapped different available genome assemblies to make them more applicable for more than one assemblies of the same species. For instance, we have mapped *Gh_HAU_v1 / v1.1 (Ghir_A11G008640)* with *Gh_CRI_v1* (*Gh_A11G085800.1*) and *Ghir_ BGI_v1* (*CotAD_01546*). Similarly, *Gra_D5_B CGP_v1.0* (*Cotton_D_gene_10022874*) with *Gra_JGI_v2.0* (*Gorai.001G109400*)^25,27,28,33–35^.

Conserved domain architecture was carried out to find duplicated domains and additional associated domains with stress-antifungal motifs^37^ protocol. The predicted domains were arranged at their specific site on amino acid sequence using the Perl program. Three different classification methods were implemented in this study; (1) types and location (N-terminal or C-terminal) of additional domains associated with the Stress-antifungal/DUF 206/Gnk2 domain with irrespective of duplicated domains, (2) complete domains, and (3) literature classifications e.g. sdCRRSP, ddCRRSP, PDLP, sdCRK, tdCRK and qdCRK^15^. We also included 35 land plants including mosses, bryophyte, gymnosperm, and angiosperm for evolutionary study of *CRKs*.

### Protein statistics, chromosomal mapping, intron–exon distribution, and motif analysis

All gene and proteins associated data were retrieved from Cottongen, including protein length, molecular weight (kDa), charges, grand average of hydropathy, isoelectric point (Ip), chromosome start, and end. The chromosomal mapping was carried with TBTools gene location, intron–exon distribution generated with gene display server^38^, structural and functional motifs were detected with the MEME motif, and PROSIT Motifs discovery server^39,40^.

### Evolution and diversity analysis of *CRKs* in *Gossypium*

An advanced comparative genomics tool, OrthoFinder^41^ was exploited to understand evolution and diversity in CRK proteins among five species. An additional DIAMOND tool was used for fast sequence similarity searches^42^. The graph clustering was done with the MCL clustering algorithm^43^. The gene tree inference and a distance matrix of the orthogroups were constructed with DendroBLAST^44^. A distance-based phylogeny tree was constructed using FastME 2.0^45^. For multiple sequence alignment, MAFTT 7.0 was used^46^. The maximum likelihood phylogenetic tree of large alignment was constructed using FastTreeMP^47^. To construct the Circos plot of five genomes, a BlastP program was used to determine the linkage and the circular plot was constructed with Advance Circos plot packages in TBTools.

### Expression profiling of *CRKs* genes

The expression profiling data is divided into three categories, *i.e.,* tissue-specific (leaf, stem, root, ovule, etc.) expression, abiotic stress-specific (cold, heat, salinity, drought) expression, and biotic stress-specific CLCuD (Cotton leaf curl virus disease). To determine the expression profiling, the publicly available RNA-seq (*SRP044705, SRP042128, SRP017168, SRP001603, SRP009820,* and *SRP027533*) at CottonFGD^48^, whitefly infestation on CLCuD susceptible accession of *G. hirsutum* (*SAMN07519654, SAMN07519653, SAMN07519652, SAMN07519651, SAMN07519650, SAMN07519649*)^49^ and whitefly infestation on CLCuD resistant accession of *G. hirsutum* (*SAMN07251316* and *SAMN07251315*)^50^ were used. The transcript level was calculated in fragments per kilo base per million (FPKM) by RNA-seq data pipelines. The gene expression clustering was carried using TBTools with parameters; log2 base, column cluster, and row cluster.

### Protein–protein interaction network and host–pathogen model docking

The *CRKs* protein–protein interaction network was generated using a STRING server with *G. raimondii* proteome interaction background. Begomovirus, a genus of the Geminiviridae family, also known as plant virus, infects a wide range of dicotyledonous plants. In cotton plants, it causes CLCuD. So, we included all CLCuD viral proteins (AV1, AV2, AC3, AC2, AC1, AC4, and C5) for their possible interaction with cotton CRKs. The sequence-based interaction was predicted using the Host–Pathogen Interaction predictor (HOPITOR)^51^. The 3D structure of CRKs and viral proteins were predicted using the I-TASSER server^52^. The host–pathogen protein docking was carried with ZDOCK^53^. The protein complex was visualized with discovery stadio^54^ and the active sites and interactive bonds were presented with Ligplot + ^55^.

### SNPs and InDels determination in CLCuD resistant and susceptible *G. hirsutum* accession

To find the genetic diversity of *CRKs* in cotton leaf curl disease-resistant and susceptible *G. hirsutum* accessions, a genome resequencing data of Mac7 (CLCuD resistant accession; developed by USDA through the breeding program) and Coker 312 (highly susceptible to CLCuD accession) was used. The resequencing data NCBI: PRJNA756435 (Mac7) and NCBI: PRJNA542238 (for Coker 312) has been used to find SNPs and InDels in the CRK genes. The raw reads of Mac7 and Coker 312 mapped to TM_1 reference gnome (*HAU-AD1_genome_v1.0_v1.1*) using a BWA aligner and followed the next-generation sequencing pipeline similar to Zhao et al.^56^ to find variant calling format files of CRK genes. The identified SNPs and InDels were annotated using the SnpEff tool with the reference genome (*HAU-AD1_genome_v1.0_v1.1*)^26^.

### Target site prediction and expression profiling of miRNA

To find the miRNA target site in CRK coding sequences of *Gossypium* sp. mature miRNA sequences were retrieved from the Plant non-coding RNA database^57^ and PmiREN^58^. These downloaded miRNA and CDS sequences of *Gossypium* sp*.* were used as input data in psRNA target: a plant small RNA target analysis^59^.

The expression profiling of CRK-targeted miRNA was assessed using the miRNA-seq of *Gossypium* sp. data located at *PmiREN* (Plant Micro RNA Encyclopedia )^58^ covering different tissues including anther, fiber, embryogenic, hypocotyl, leaf, ovule, root, shoot apical, stem, and apexes.

### Plant growth and CLCuD stress

To validate the RNA-seq data of *GhCRKs*, we selected two *G. hirsutum* accessions, Mac7 (resistant to CLCuD) and Coker 312 (susceptible to CLCuD). A set of 20 plants was sown in the glasshouse for each accession. After five weeks of post-germination, one set of each accession (10 plants) was transplanted in the net house to expose the whitefly (CLCuD career vector). After two weeks of post-transplantation, a high population of whitefly was seen on Coker 312’s as well as Mac7 plants. All Coker 312 were 100% infected with severe symptoms, while no symptom was found in Mac7 plants.

### RNA extraction and real-time quantitative PCR analysis

Young leaves were collected from net house and glasshouse. Total RNA was extracted through the Trizol method^60^, and treated with RNase-free DNase (Promega, USA). The quality was assessed by gel electrophoresis. A 12μL sample with 100 ng/μL concentration, converted into cDNA using RevertAid Hminus First Strand cDNA Synthesis Kit (Thermo Scientific).

Based on the results of biotic stress expression profiling and host–pathogen protein interaction of *GhCRKs*, a set of genes was selected for qPCR analysis, and gene-specific primers were designed. Real-time PCR was performed using a Bio-Rad iCycler Thermal Cycler iQ5 and DNA Master SYBR Green I kit (Roche, Basel, Switzerland). Reactions were carried out in triplicate and each replicate consisted of 2 μL of cDNA (with concentration of100ng/μL), 0.5μL of each primer (concentration 10 μM/μL) and 5 μL SYBR Green Master Mix, making a final volume of 12 μL reaction. The PCR reactions were carried out using the following conditions: the initial temperature at 95 °C for 5 min, followed by 35 cycles of 95 °C for the 30 s, 58 °C for 30 s, and 72 °C for 1 min. Each biological sample was used in triplicates and the average expression value was calculated. The data ware normalized with the largest value of each panel making the highest relative expression as one.

## Results

### Gene organization of *CRKs* in diploid and tetraploid cotton species

**Figure 1:**
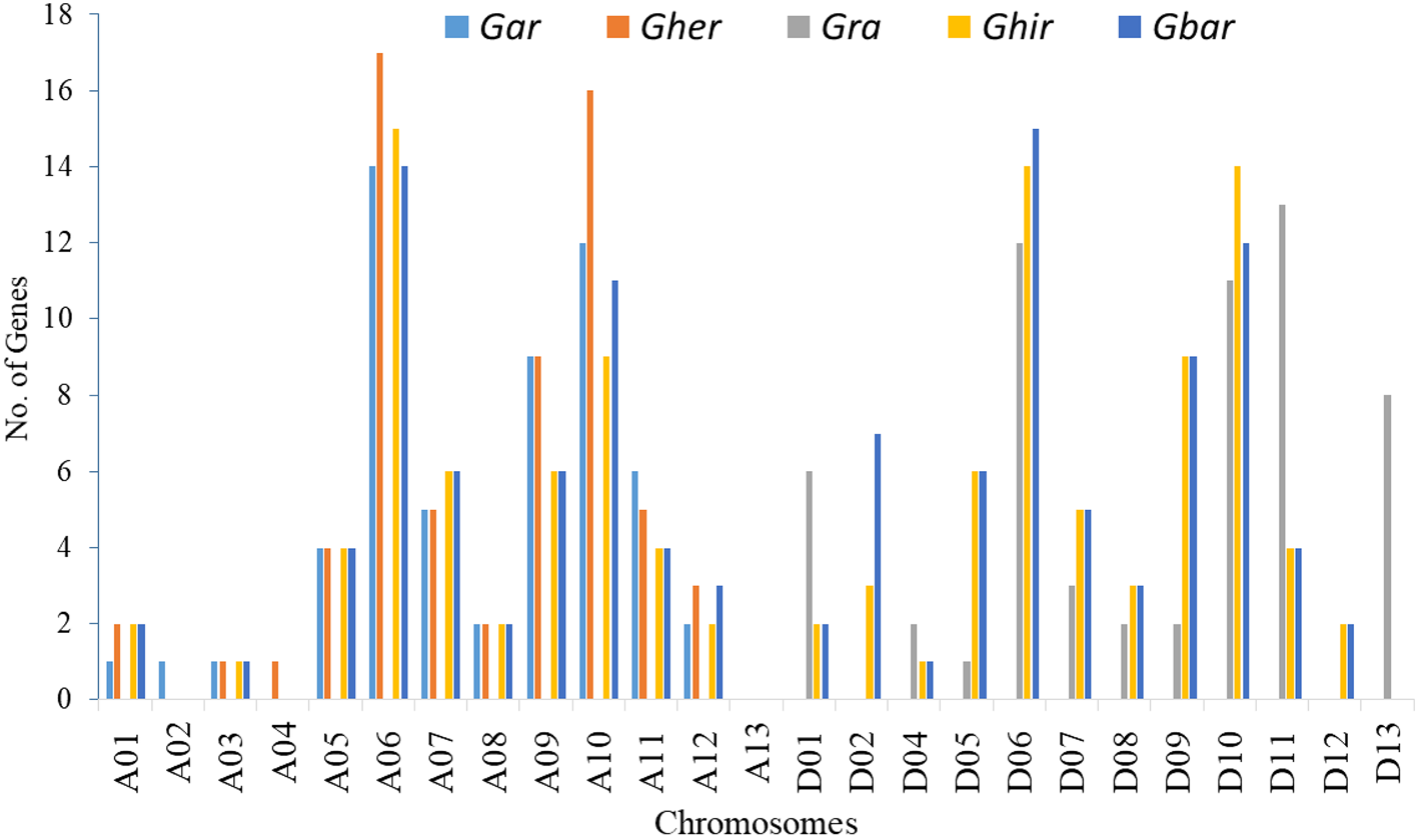
Gene density on A-like and D-like genomes of *Gossypium* sp*.*

The genome-wide analysis identified a total of 60, 74, 65, 120, and 118 CRK genes in *Gar, Gra, Gher, Gbar,* and *Ghir*, respectively (Tables S1–S5). The protein features were presented for each species, including protein length, molecular weight, charge, isoelectric point, and grand average of hydropathy. A summary of these features showed that the longest proteins sequence comprises 884 amino acids, while the shortest was composed of only 127 amino acid residues. Likewise, the molecular weight range was observed between 97.675 and 14.55 kDa, whereas the protein charge ranges between + 23.5 and  − 15. Moreover, the isoelectric point ranged from 9.229 to 4.426, while the grand average of hydropathy range was from 0.172 to − 0.347 (Fig. S1).The chromosomal location and frequency of genes among the A-genome, D-genome, A-like genome, and D-like genome also demonstrated nearly similar gene density on respective chromosomes. For instance, the maximum number of genes was localized on Chr6, Chr10, Chr11 in the D-genome (*Gra*), and A-genome (A1; *Gher*, A2; *Gar*). Similarly, ChrA06 (*Gar;* 14 genes*, Gher;* 17*, Ghir*; 15 genes and *Gbar* with 14 genes), ChrD06 (*Gra*; 12 genes*, Ghir*; 14 genes*,* and *Gbar*; 15 genes), ChrD09, and ChrD10 (*Gra;* 11*, Ghir*; 14*,* and *Gbar;* 12 genes) possessed the highest number of genes in respective species. Additionally, most of the genes were found in clusters and were localized on the terminal arms of chromosomes. The gene clusters were randomly distributed along centromeres and telomeres (Fig. 1, Table S6). Overall, we found that most of the genes were localized on Chr6, Chr10, and Chr11 in all five species, representing their common locus the genomes.

### Domain architecture, classification, and phylogenetic analysis of cotton *CRKs*

**Figure 2:**
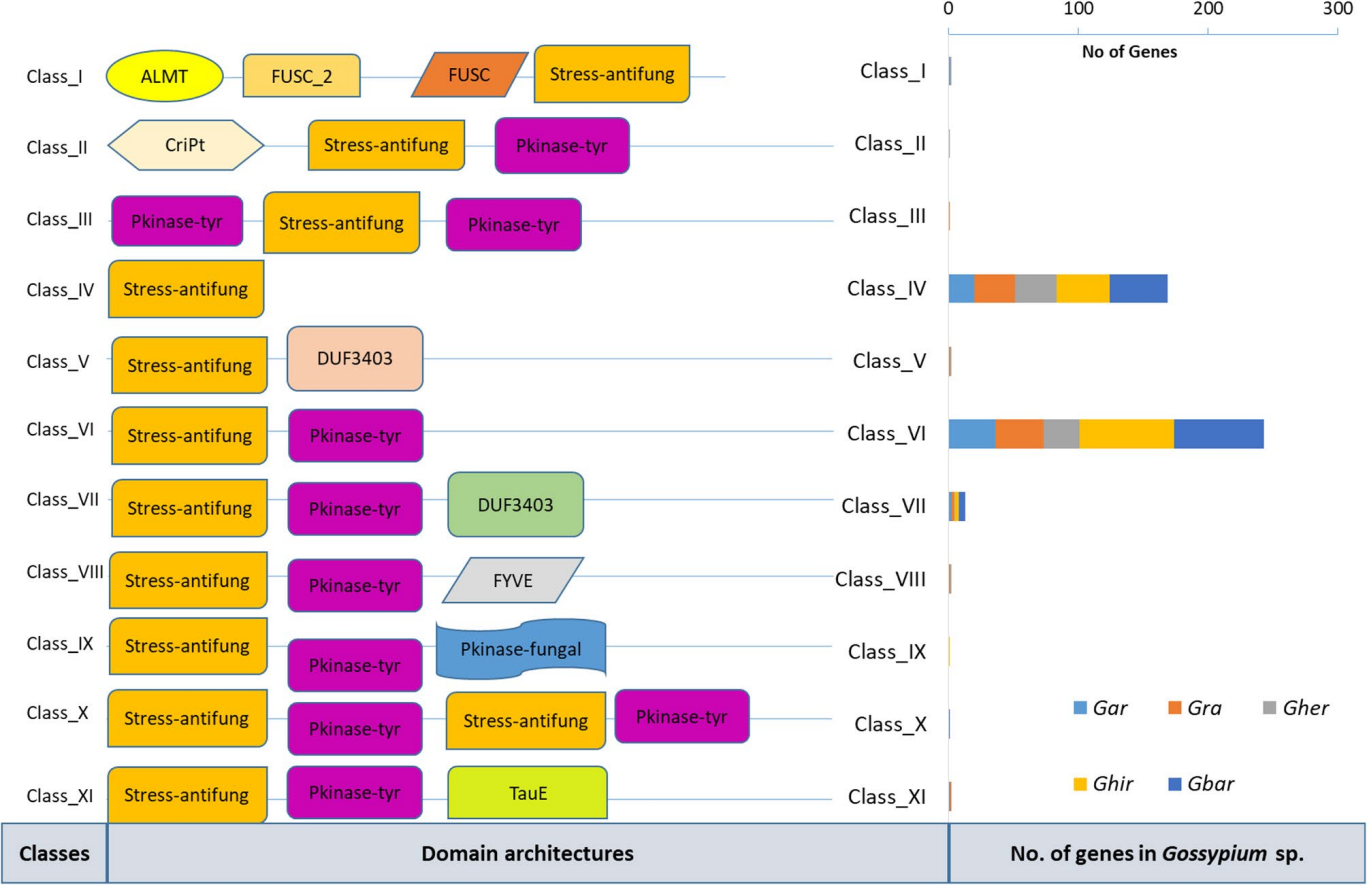
Major classes of *CRKs* based on 1st classification method and their number of genes in five species G. *arboreum* (*Gar*), *G. raimondii* (*Gra*), *G. herbaceum* (*Gher*), *G. hirsutum* (*Ghir*), and *G. barbadence* (*Gbar*).

To provide a comprehensive study, we have introduced two new classification methods in cotton *CRKs*. The first was based on the type of domains presence and absence. In this classification system, all identified CRK genes were divided into 11 major classes. Through this classification, we have identified many Stress-antifungal (DUF 26)-associated functional domains such as *ALMT, FUSC_2, Cript, DUF3403, FYVE, TauE*, and *Pkinase-tyr*. Of these 11 classes, class VI (*Stress-antifungal—Pkinase-tyr*) has the largest number of genes, followed by class IV and VII. Class VI was the most commonly found class in all species with 73, 69, 36, 28, and 37 genes in *Ghir, Gbar, Gar, Gher,* and *Gra*, respectively. In contrast to shared classes, several species-specific classes (*e.g.* class_II only found in *Gher*, class_II only in *Gra*, class_IX only in *Ghir,* and class_X in *Gbar*) were also observed (Fig. 2, Table 1, Table S7).

**Table 1:** Major classes of cotton CRKs. The classification is based on absence and presence of additional functional domains.

Major Classes	Domain architectures	Total no. of genes
Class_I	*ALMT–FUSC_2–FUSC–Stress-antifungal*	2
Class_II	*Cript–Stress-antifungal–Pkinase-tyr*	1
Class_III	*Pkinase-tyr–Stress-antifungal–Pkinase-tyr*	1
Class_IV	*Stress-antifungal*	169
Class_V	*Stress-antifungal–DUF3403*	1
Class_VI	*Stress-antifungal–Pkinase-tyr*	2
Class_VII	*Stress-antifungal–Pkinase-tyr–DUF3403*	243
Class_VIII	*Stress-antifungal–Pkinase-tyr–FYVE*	13
Class_IX	*Stress-antifungal–Pkinase-tyr–Pkinase_fungal*	2
Class_X	*Stress-antifungal–Pkinase-tyr–Stress-antifungal–Pkinase-tyr*	2
Class_XI	*Stress-antifungal–Pkinase-tyr–TauE*	1

The second classification method included the number of duplicated domains in addition to domain presence and absence. These classes were named as a subclass of CRK genes in *Gossypium* sp. All 437 genes were distributed into 43 sub-classes (I- XXXIII classes). The highest number of genes were observed in subclass IX (162 genes) with domain architecture *Stress-antifungal__Stress-antifungal__DUF3403* and VII subclass (116 genes) with domains; *Stress-antifungal__Stress-antifungal.* These two classes were commonly found in all five species with the highest number of genes compared to other subclasses. In contrast to common domains architectures, unique and species-specific domain architectures were also observed (Fig. S2, Tables S8–S9).

The accumulative phylogenetic tree of all five species is divided into several major and minor clades. However, we did not observe any species-specific clade showing species diversity in the *CRKs* among *Gossypium* sp. The whole phylogenetic tree was divided into 51 subclades/clusters (clus), and of these, clus-XLVII had maximum genes with 15 *CRKs*, followed by clus-V, clus-V, clus-XXIII, and clus-VII with 14 CRKs. Similarly, clus-I, clus-XIV, and clus-XLIII consist of 13 *CRKs* and so on. Most of the clades possessed a range of 7 to 14 *CRKs*. The number of clades was related to subclasses of cotton *CRKs* (Fig. S3). Taken together, these *CRK* classifications identified several novel classes of species-specific and common members.

### Evolutionary study of cotton *CRKs* with land plants

**Figure 3:**
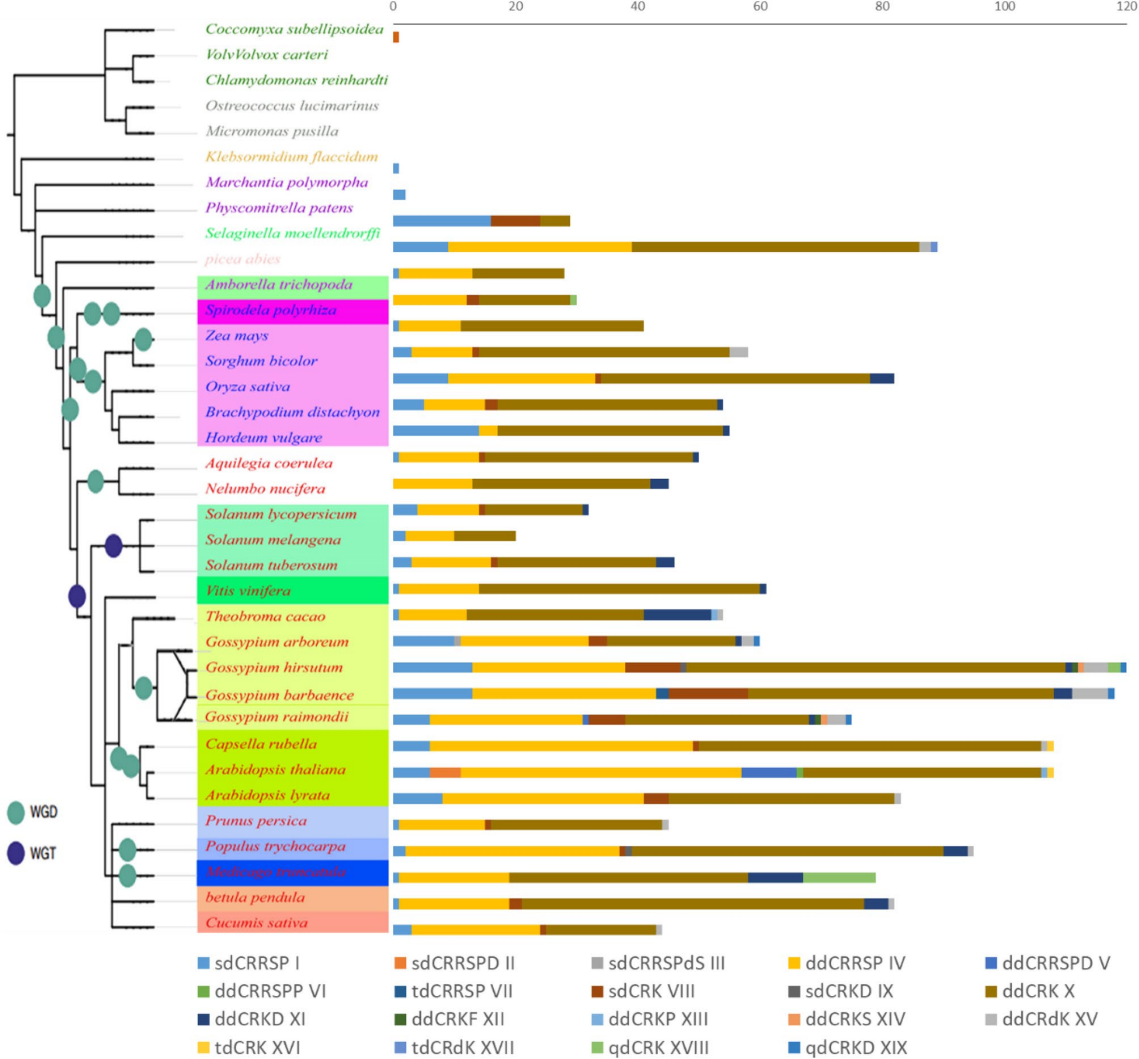
Land plants species phylogenetic tree with their total no. of *CRKs* and classes. The roman number are presenting classes based on 3rd classification system.

For the evolutionary study of *Gossypium* sp*.* with land plants, we included 2,026 *CRKs* from 35 plants, including mosses, bryophyte, gymnosperm, and angiosperm^15^ (Table S10). Of these land plants, *Chlamydomonas reinhardtii, Coccomyxa subellipsoidea, Micromonas pusilla, Ostreococcus lucimarinus, Volvox carteri* did not possess any CRK encoding genes. The identified *CRKs* were screened through *Pfam* and revised the steps mentioned for cotton classification. The conserved domain-pattern-based classification identified a total of 19 different patterns, thus classified into 19 subclasses (Table 2). Of these nineteen classes, class IV (ddCRRSP) and class X (ddCRK) were more common in all higher plants, while lower plants e.g.,* Marchantia polymorpha* (liverwort) had only class I (sdCRRSP) followed by *Selaginella moellendorffii* (lycophyte) that possessed class I, class VIII (sdCRK) and class X (ddCRK). The *Gossypium* sp. showed several genus-specific classes including sdCRRSPdS, ddCRRSPD, tdCRRSP, sdCRKD, ddCRKF, ddCRKS and qdCRKD. These classes are only found in cotton species showing their diversity with other dicot plants (Fig. 3, Table S11).

**Table 2:** Classes of CRKs, based on the number of stress-antifungal (DUF 26) domains, found in 35 land plants.

Class no	Class Name	Functional domains	Total no. of genes
I	sdCRRSP	Single stress-antifungal domain	143
II	sdCRRSPD	sdCRRSP with N-terminal DUF domain	5
III	sdCRRSPdS	sdCRRSP with double sugar transporter domain	1
IV	ddCRRSP	Double stress-antifungal domain	521
V	ddCRRSPD	ddCRRSP with N-terminal DUF3403 domain	10
VI	ddCRRSPP	ddCRRSP with N-terminal PRIMA1 domain	1
VII	tdCRRSP	Triple stress-antifungal domain	2
VIII	sdCRK	Single stress-antifungal domain with N-terminal kinase domain	58
IX	sdCRKD	dCRK with N-terminal DUF3403 domain	2
X	ddCRK	Double stress-antifungal domain with N-terminal kinase domain	947
XI	ddCRKD	ddCRK with *DUF3403* domain	49
XII	ddCRKF	ddCRK with N-terminal *FYVE* domain	2
XIII	ddCRKP	ddCRK with N-terminal *PRIMA1* domain	2
XIV	ddCRKS	ddCRK with N-terminal sugar transporter domain	2
XV	ddCRdK	ddCRK with special fungal *kinase* domains	27
XVI	tdCRK	Triple stress-antifungal domain with single kinase domain	2
XVII	tdCRdK	Triple *stress-antifungal* domain with single kinase domain and special fungal kinase domains	1
XVIII	qdCRK	Tetra *stress-antifungal* domain with single kinase domain	15
XIX	qdCRKD	Tetra stress-antifungal domain with single kinase domain and *DUF3403* domain	4

**Figure 4:**
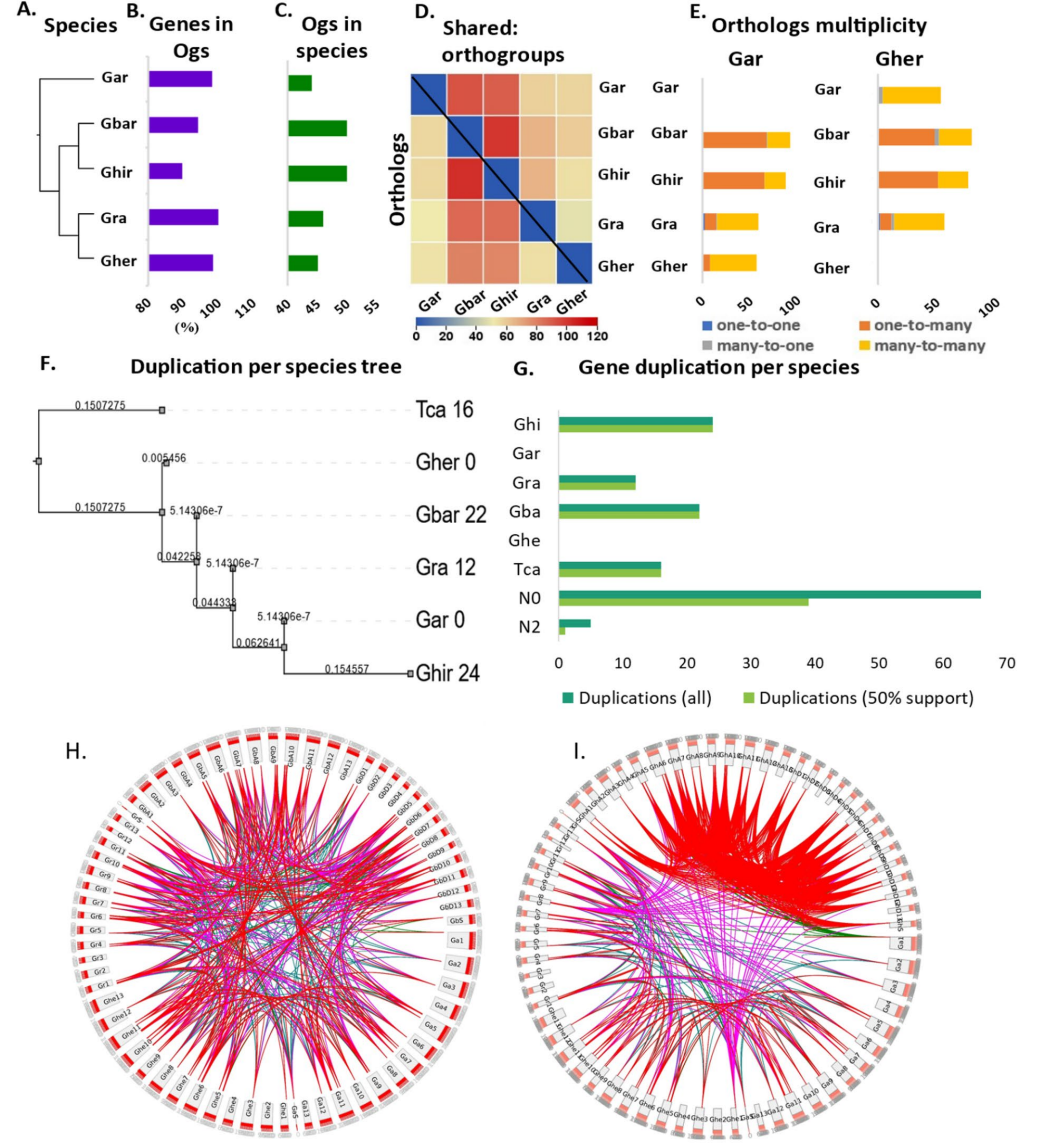
Summary of OrthoFinder analysis of CRK gene family. (**A)** The species of *Gossypium* sp**. (B)** Percentage of genes in orthogroups. (**C)** The number of species-specific orthogroups. (**D)** Heat map showing shared orthogroups. (**E)** Orthologs multiplicity. (**F)** Gene duplication events per species. (**G)** Gene duplication per internal and terminal nods of the species-based-phylogenetic tree. (**H**) Circos plot of *Gar, Gher, Gra* with *Gbar*. (**I)** Circos plot of *Ghir* with *Gar, Gher* and *Gra*. *Tca*; *T. cacao.*

The comparative genomics summarized that all *CRKs* from 5 species of cotton plants were divided into 52 orthogroups covering 416 genes (95.2% of genes in orthogroups) with only 21 unassigned genes (4.8% of genes). Of these, 40 orthogroups were shared by all five species, while none of the orthogroups were species-specific. Overall, the mean and medians were recorded as 8 and 7 orthogroups, respectively. At the species level, however, we observed that *G. arboreum* shared more orthologs with *G. barbadense* (92 orthologs) and *G. hirsutum* (89 orthologs) as compared to *G. raimondii* (58 orthologs) and *G. herbaceum* (57 orthologs). Similarly, *G. barbadense* shared a higher number of orthologs with *G. hirsutum* (100 orthologs), followed by *G. raimondii* (66 orthologs), *G. arboreum* (56 orthologs), and *G. herbaceum* (54 orthologs) (Fig. 4A–C).

The species-wise orthogroups distribution represented that most of the identified genes belonged to one of 52 orthogroups, i.e., *G. arboreum* (84.6% of total *GaCRKs*), *G. raimondii* (88.5% of total *GrCRKs*),) and *G. herbaceum* (86.5% of total *GheCRKs*), *G. barbadense (*84% of total *GbCRKs*), and *G. hirsutum* (96.2% of total *GhCRKs*). The species-wise relationship demonstrated remarkable relation among five species. We also drew one-to-one, one-to-many, many-to-one, and many-to-many species relationships. We concluded that a small number of genes contributed in one-to-one and many to one, while a higher number of genes showed one-to-many and many-to-many with a concluding close relationship of five species (Fig. 4D,E).

The species-wise phylogenetic tree with *Theobroma cacao* (*T. cacao*) as an outgroup, demonstrated that *G. arboreum* (A2-genome) has close relation with *G. hirsutum*, followed by *Gra* (D-genome). The gene duplication event was also predicted at all internal and terminal nodes. N_0_ node represented the common ancestor of all cotton species with *T. cacao*, demonstrated 69 duplications with 100% confidence and 40 duplications with 50% confidence. Emerging from the N_0_ node, *T. cacao* gained 16 duplications, *Gbar* gained 22, *Gra* gained 12 and *Ghir* gained 24 duplication events. While the *Gher* (A1-genome) and *Gar* (A2 genome) did not show any duplication events. (Fig. 4F,G). The chromosomal location collinearity suggested that the *Ghir* has more syntenic blocks within its subgenome (A and D genomes) followed by *Gar* (A-genome) while the *G. barbadence* showed more collinearity lines with *Gher* (A1-genome) and *Gra* (D-genome) (Fig. 4H,I).

### De-novo motif discovery and functional sites prediction and tissue-specific expression profiling in diverse cotton species

The de-novo MEME motif analysis identified a total of 15 conserved motifs in all-cotton CRK genes (Fig. S4). Of these, motif_13, motif_9, motif_2, and motif_12 are highly conserved in all *CKRs*. However, motif_1, motif_4, motif_11, and motif_6 were gene-specific. In addition, the functional motif sites prediction through *PROSITE* identified, a total of 26 important functional motifs including *asn_glycosylation, myristyl, ck2_phospho, rgd, pkc_phospho, protein_kinase_dom, protein_kinase_atp, camp_phospho, protein_kinase_st, amidation, leucine_zipper, hma_1, phe_rich, atp_gtp_a, ser_rich, n6_mtase, pro_rich, tonb_dependent_rec_1, peroxidase_1, prokar_lipoprotein, zf_fyve, microbodies_cter* and *prenylation*. Overall, we concluded that the different groups of *CRKs* possessed different functional and structural motifs that might provide functional diversity (Table S12).

The RNA-seq data analysis of CRK genes in *G. arboreum* showed distinctive expression patterns in diverse tissues (leaf, stem, and root) at different time intervals (10 DPA, 15 DPA, and 20 DPA). Some genes, including *GaCRK02, GaCRK09, GaCRK52,* and *GaCRK29,* showed increased transcript levels in the leaf than stem or root. Similarly, several genes (*GaCRK02, GaCRK24, GaCRK03,* and *GaCRK38*) had elevated transcript levels in the stem, while others exhibited higher mRNA levels in the root (*GaCRK24, GaCRK02, GaCRK43,* and *GaCRK07*). In addition, the expression profiling in the ovule at 10 DPA, 15 DPA, and 20 DPA demonstrated remarkable differences in the expression of CRK genes. For instance, in ovule development at 15 DPA, *GaCRK03, GaCRK02,* and *GaCRK38* showed the highest expression levels, and this set of genes also showed similar results at 15 DPA and 20 DPA in ovule and fiber development (Fig. S5A, Table S13). In summary, most of the genes demonstrated tissue-specific expression, However, *GaCRK02* showed high transcripts in all tissues at different time intervals.

The *G. raimondii* RNA-seq analysis of *CRKs* in different tissues such as seed, fiber, ovule, and leaf at different time intervals (10 DPA, 20 DPA, 30 DPA, and 40 DPA) was also presented. Results demonstrated that *GrCRK11, GrCRK67,* and *GrCRK52* showed higher transcript levels in seed germination at 10 DPA in comparison with 20 DPA, 30 DPA, and 40 DPA data. Similarly, some genes (*GrCRK67, GrCRK21, GrCRK24,* and *GrCRK42*) exhibited increased transcript levels at 20 DPA, while others showed higher mRNA levels at 30 DPA (*GrCRK21, GrCRK67, GrCRK24,* and *GrCRK42*) and 40 DPA (*GrCRK63, GrCRK21, GrCRK11,* and *GrCRK67*). Similar expression patterns were also observed in fiber development at 10 DPA (*GrCRK24, GrCRK11, GrCRK46,* and *GrCRK18*) and 20 DPA (*GrCRK06, GrCRK21, GrCRK67,* and *GrCRK24*) (Fig. S5B, Table S14). Like the *G. arboreum* CRKs expressions, the *G. raimondii CRKs* also demonstrated clusters of genes co-expressing in different tissues. However, *GrCRK67* showed its putative role in all tissues.

**Figure 5:**
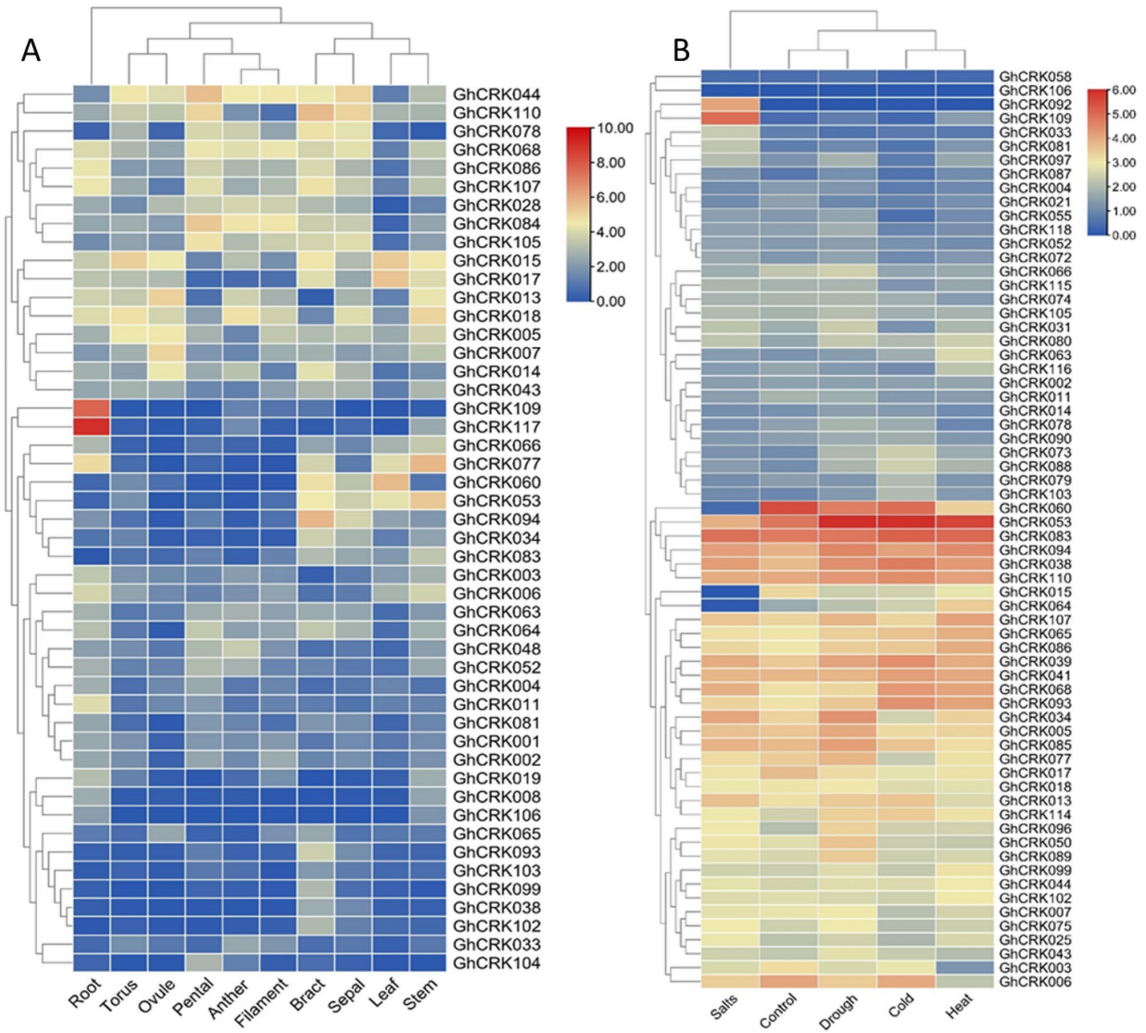
Expression profiling of *GhCKRs*. (**A)** in different tissues, (**B)** under different abiotic stresses.

*Gossypium hirsutum* is known as upland cotton, and these species produce more than 90% of the world's raw cotton. Therefore, several transcriptomics data are available for this plant. The tissue-specific RNA-seq data included different tissues (leaf, bract, sepal, stem, root, ovule, torus, filament, petal, and anther). The RNA-seq results demonstrated diverse tissue-specific expression patterns of CRKs in various tissues. Some prominent genes (*GhCRK044, GhCRK110, GhCRK068, GhCRK086, GhCRK107,* and *GhCRK084*) exhibited high transcript values in filament, petal, and anther. However, a few genes associated with similar clusters depicted higher expression in stem (*GhCRK77, GhCRK053, GhCRK013, GhCRK015,* and *GhCRK018*), ovule (*GhCRK007, GhCRK013, GhCRK015, GhCRK014*, and *GhCRK005*), torus (*GhCRK*018, *GhCRK*015, *GhCRK*005, and *GhCRK*044) and in bract (*GhCRK094, GhCRK053, GhCRK060, GhCRK077, GhCRK110*, and *GhCRK*078) tissue. These differential expressions indicate the role of CRK genes in the growth and development of multiple tissues (Fig. 5A, Tables S15–S16).

The RNA-seq data of CRK genes under different abiotic stresses including salt, drought, heat, and cold stresses was demonstrated at different time intervals (1 h, 3 h, 6 h, and 24 h) and days intervals (0 DPA, 1 DPA, 3 DPA, 10 DPA, 15 DPA, and 20 DPA) (Tables S17–S21). The comparative expression profiling revealed several differentially expressed gene clusters displaying increased transcripts values at corresponding stress conditions. For instance, a cluster of genes including *GhCRK060, GhCRK053, GhCRK083,* and *GhCRK110* showed high expression under all stresses including salt, drought, heat, and cold. However, most of the *CRKs* depicted tissue-specific expressions. For instance, *GhCRK109* was highly expressed under salt stress while did not show induced in other stresses. Similarly, the *GhCRK053* gene is highly induced under drought, cold and heat stresses rather than salt stress. The clusters of genes that showed their co-expressions and co-occurrences under specific stresses might have an accumulative role in cotton plant adaptation during environmental stresses (Fig. 5B).

### Protein–protein interaction network and host–pathogen interaction

**Figure 6:**
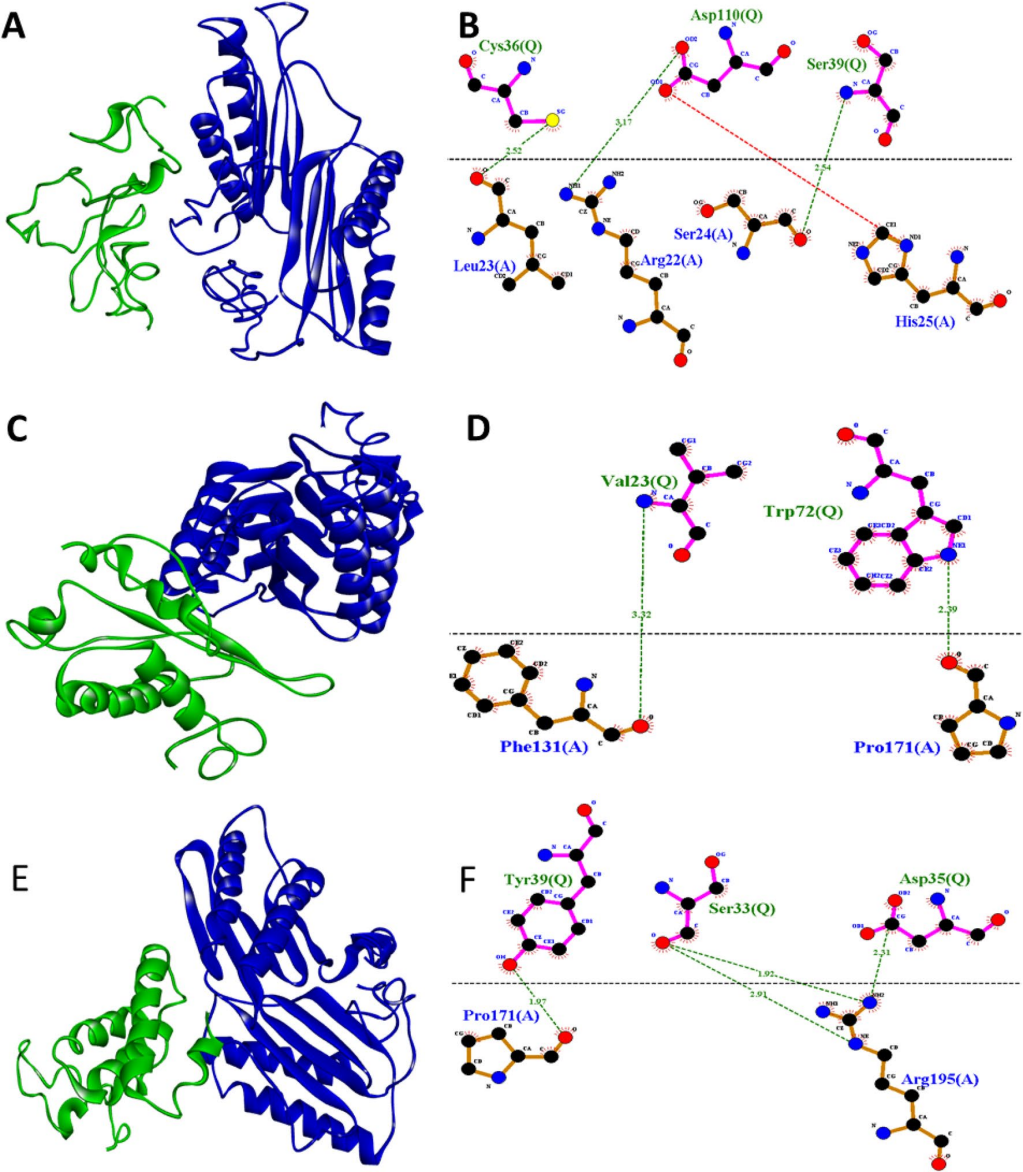
Molecular docking of host–pathogen interaction. (**A)** *GhCRK21-AC2* complex, (**B)** *GhCRK21-AC2* complex 2D interaction graph, (**C)** *GhCRK45-AC3* complex, (**D**) 2D interaction graph of *GhCRK45-AC3* complex,(**E**) *GhCRK45—V2* complex, (**F**) 2D interaction graph of *GhCRK45—AV2*. The green color represents viral proteins and blue is for Host CRKs. Green lines; hydrogen bond, red lines; salt bridge.

The protein–protein interaction network of *GhCRKs* provided endogenous protein interactions including, experimentally determined interactions, gene fusion, gene co-occurrence, co-expression, and protein homology. Of 118 *GhCRKs*, only a few proteins showed internal interactions. For instance, *GhCRK067* has the highest number of interactions including *GhCRK067-GhCRK025* and *GhCRK67-GhCRK048* possessing experimentally validated interactions, *GhCRK067-GhCRK028* complex has three types of correlation *i.e.* Co-expression, protein homology, and text mining. Similar interactions were also observed in the *GhCRK084-GhCRK028* complex (Fig. S6). We selected ten genes for further host–pathogen interaction analysis based on the differential expression of *CRKs* in Mac7 and NIAB-Karishma under CLCuD stress. The protein–protein interaction probability analysis demonstrated strong interaction of most of the up-regulated genes in Mac7 to the Begomovirus protein, including *GhCRK082* (strongly interaction probability with *AC1, AC2, AC3, AC4, AV2,* and *C5*) and *GhCRK087* (strongly interaction probability with *AC1, AC2, AC3,* and *AV2*) had a probability value of more than 0.9. In comparison, almost all other genes had greater than 0.5 values, which is significant for protein–protein interaction (Fig. S7, Table S22). Furthermore, the sequence-based interactions of *GhCRKs* with viral proteins were also demonstrated with host–pathogen protein–protein interaction with ZDOCK molecular docking. The 3D host–pathogen protein docking analysis demonstrated the interaction network between host and pathogen amino acidic residues. The upregulated genes and their interaction with CLCuD viral proteins confirmed their direct interaction. The *GhCRK059* and *AC2* interaction provided the active residues and their bonding types. As such, the *GhCRK059* protein (A chain) with residues Leu_23_, Arg_22_, Ser_24,_ and His_25_ interacted with *AC2* protein (Q chain) at Cys_36_, Asp_110,_ and Ser_39_ through hydrogen bond (green lines) and salt bridge (red lines). Similar results were observed in *GhCRK087-AC3*, *GhCRK087-AV2*, *GhCRK082-AC2, GhCRK082-AV2*, and *GhCRK082-AC3* complexes. However, the number of bonds and types of bonds varied from complex to complex e.g., the highest number of interactions was found in *GhCRK082-AC3*, followed by *GhCRK082-AC2* (Fig. 6, Fig. S8).

### SNPs and InDels variants in *CRKs* of resistant *G. hirsutum*

Mac7 is a tolerant *G. hirsutum* accession, which is developed by USDA by breeding program. To find genetic variation and transcriptomics variation, we have identified SNPs and InDels associated with *CRKs* in Mac7 and Coker 312. The genome-wide-genetic variation in Mac7 identified a total of 192 and 208 genes having SNPs and InDels concerning TM-1 reference *G. hirsutum* genome, respectively. Similarly, a total of 62 and 192 genes with SNPs and InDels were found in Coker 312, respectively. The comparative study of Mac7 and Coker 312 identified several unique SNPs and InDels in different genomics regions with different effects. For instance, 82 nonsynonymous, 64 synonymous, and 51 in 3’ UTR, SNPs were observed Mac7 (Table S23). In addition to genomic region-based variants, we also categorized variants into impact-based levels *e.g.* high, low, moderate, and modifier. In the case of variants by impacts, a total of 12 and 23 genes showed high impact SNPs and InDels in Mac7, respectively. While only 3 SNPs and 17 InDels associated genes were found in Coker 312 under the same variant impact level (Table S24). In summary, we have observed many differences in CRK gene sequences of the Mac7 and Coker 312 (Fig. S9). There were several unique SNPs and InDels in CRK genes of Mac7 accessions which could be the source of resistance to different biotic and abiotic stresses.

### Micro-RNA and their target sites prediction in *CRK* genes

The miRNA target site prediction analysis demonstrated that most of the CRK genes possess miRNA target sites. However, the five species understudy showed somehow common and unique miRNA families. To provide deep analysis, the CRK gene-targeted miRNA, all identified miRNA target sites were categorized into family-based and family-member-based in all five species. A total of 30, 2, 1, 117, and 30 miRNA families were detected in CRK genes of *G. arboreum* (*Gar*)*, G. raimondii* (*Gra*)^61^*, G. herbaceum* (*Gher*)*, G. hirsutum* (*Ghir*)*,* and *G. barbadense* (*Gbar*)^62^, receptively. In these miRNA families, a total of 83, 3, 1, 346, and 150 miRNAs were detected in *Gar, Gra, Gher, Ghir,* and *Gbar*, respectively. In *Gar*, some miRNA families, including *Gar-miR172* (59 target sites, *Gar-miR396* (22 target sites), and *Gar-miR1373* (14 target sites), have a high number of target sites in GaCRK genes. Of the *Gar-miR172* family, *Gar-miR172c* (7 target sites), *Gar-miR172d* (7 target sites), and *Gar-miR172e* (7 target sites) had a higher number of target sites (Fig. S10A, Table S25). In the *G. raimondii* CRK gene, only two miRNA families were detected, including *Gra-miR172 (*16 miRNA target sites*)* and Gra-miR482 (3 miRNA target sites). (Fig. S10B, Table S26). In *G. herbaceum*, only a single miRNA was detected (Fig. S10C, Table S27). Since the number of *CRKs* is higher in tetraploid cotton, we found an increased number of miRNAs. A total of 10 miRNA families were predicted in *G. hirsutum* with the highest number of target sites by *Ghi-miR172 (*296 miRNA target sites), *Ghi-miR394* (60 miRNA target sites), and *Ghi-miR1404 (58* miRNA target sites), and so on*.* At the family member level in the *Ghi-miR172* family, three major targeting members were observed as *Ghi-miR172d* (22 miRNA target sites), *Ghi-miR172e* (22 miRNA target sites), and *Ghi-miR172f* (22 miRNA target sites) (Fig. S10D, Table S28). Similarly, *G. barbadense*, *Gba-miR172, Gba-miR156,* and *Gba-miR395* families had the highest number of miRNA targets sites within CRK genes (Fig. S10E, Table S29). The comparative study of miRNA families and members among five species demonstrated that both common and species-specific miRNAs. The most common miRNA family in CRK genes was *miR172* and *miR*156 among all species. However, we also observed some species-specific miRNA families. For instance, *Ghir* had 85 unique miRNA families, *Gar* had seven unique miRNA families, and *Gbar* had only seven (Fig. S11A,B). Similar findings were also observed in miRNA family members among the five species. Overall, cotton CRK genes possessed more miRNA target sites for the miR172 family that might be the main functional regulator of cotton CRK genes.

### Expression profiling of identified miRNA families

The expression profiling of miRNA families and their members provides significant information about the CRK genes regulation. Thus, we have identified the expression level of miRNA families in different tissues of *G. arboreum, G. barbadense,* and *G. hirsutum*.

**Figure 7:**
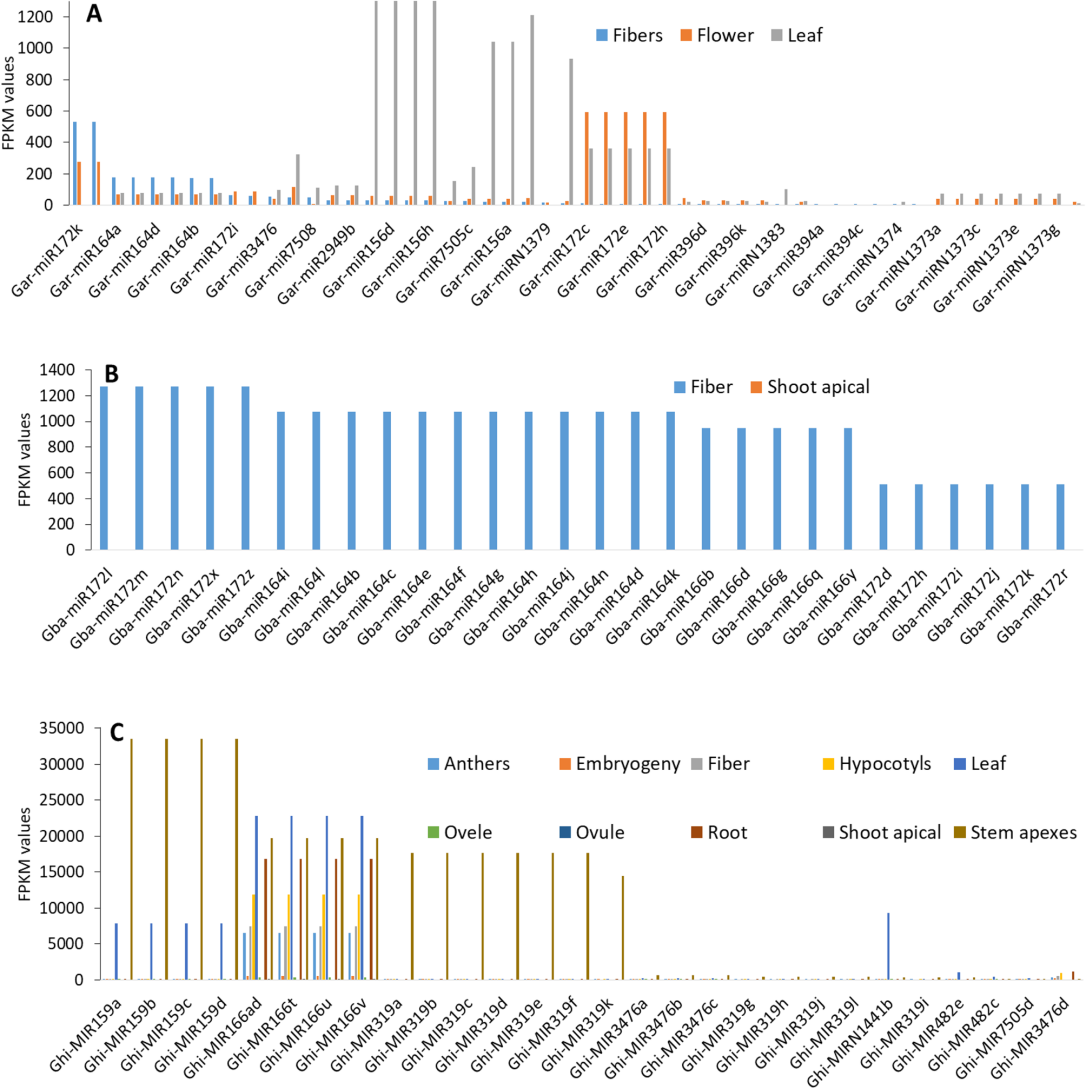
Identified miRNA family member’s expression profiling in different tissues. (**A**) *G. arboreum,* (**B**) *G. barbadense*, and (**C**) *G. hirsutum.*

For the expression profiling of *G. arboreum* miRNA, we included fiber, flower, and leaf tissues. In fiber tissue, the highest expression of *Gar-miR172, Gar-miR164,* and *Gar-miR3476* was observed. Similarly, *Gar-miR172, Gar-miR535,* and *Gar-miR164* showed elevated expression in flower, while *Gar-miR156, Gar-miR172,* and *Gar-miR535* displays increased expression in leaf (Fig. 7A, Table S30). In *G. barbadense*, we only found data for two tissues, *i.e*., fiber and apical shoot. The highest expression of miRNA (*Gba-miR172, Gba-miR164,* and *Gba-miR166)* was observed in fiber, whereas *Gba-miR156, Gba*-*miR166,* and *Gba-miR172* were found to be elevated in the shoot apical (Fig. 7B, (Table S31). In summary, we discovered a strong role of *miR172, miR156,* and *miR159* in regulating CRKs in *G. barbadense* plants. The expression profiling of *G. hirsutum* miRNA included different tissues, and most of the miRNAs showed tissue-specific expression. For instance, highest expression of miRNAs was observed in anther (*Ghi-miR166, Ghi-miR172,* and *Ghi-miR156)*, embryogenic (*Ghi-miR156, Ghi-miR166,* and *Ghi*-*miR164*), fiber (*Ghi-miR166, Ghi-miR164,* and *Ghi-miR3476*), hypocotyls (*Ghi-miR156, Ghi-miR166,* and *Ghi-miR3476*), leaf (*Ghi-miR166, Ghi-miR1441, Ghi-miR159,* and *Ghi-miR156*), ovule (*Ghi-miR166, Ghi-miR1441,* and *Ghi-miR159*), root (*Ghi-miR156, Ghi*-*miR166,* and *Ghi-miR1383*), apical shoot (*Ghi-miR156*) and in apexes stem (*Ghi-miR159, Ghi-miR166,* and *Ghi-miR319*) (Fig. 7C, Table S32). In conclusion, we have observed that *miR172* has high expression in most of the tissues like fiber, flower, and apical shoots, displaying its role in the regulation of the CRK gene.

### Expression profiling and RT-PCR analysis of GhCRKs under CLCuD in resistant and susceptible *G. hirsutum*

**Figure 8:**
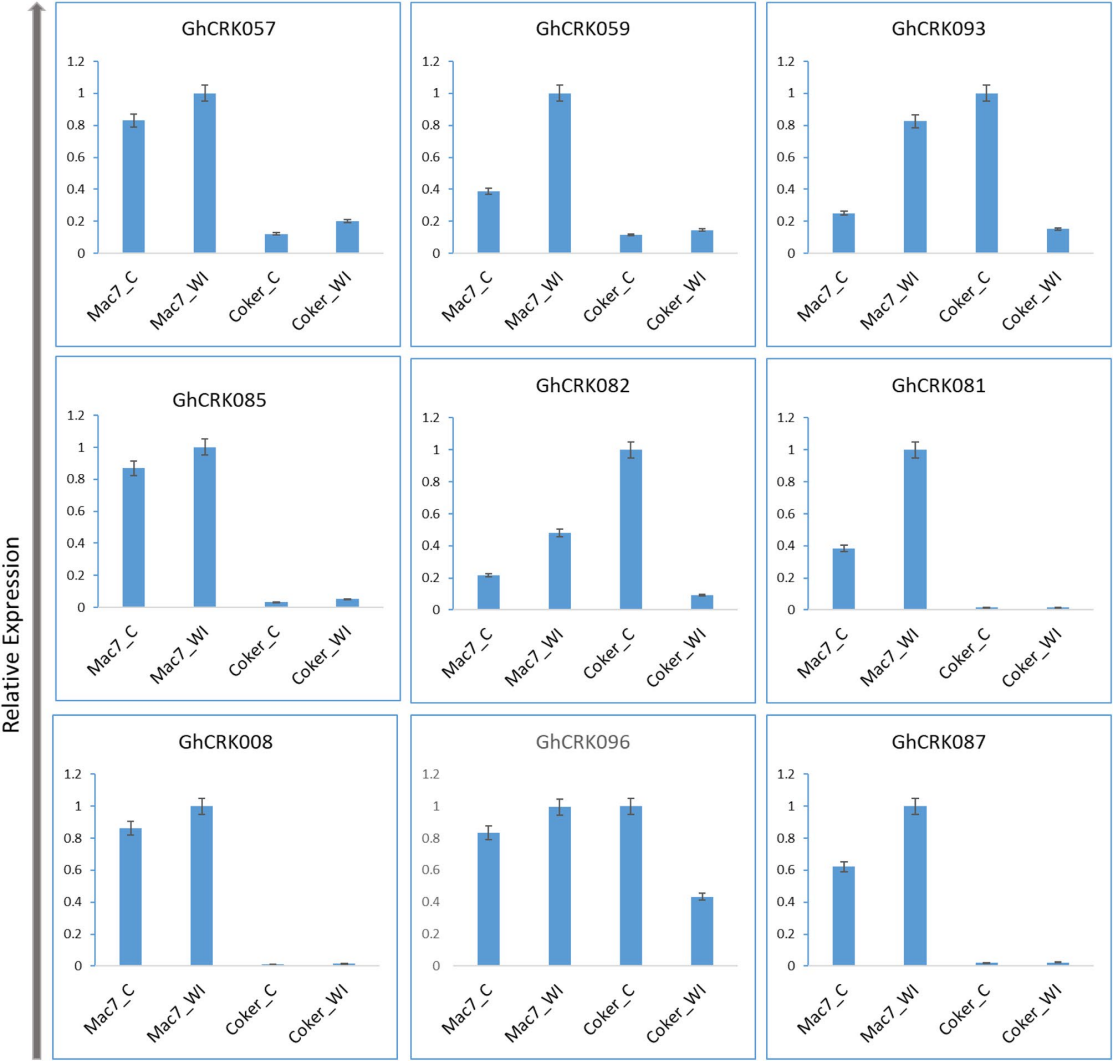
Relative expressions of *GhCRKs* under cotton leaf curl disease. Mac7_C; resistant control, Mac7_WI; resistant infested with viruliferous whitefly, Coker 312_C; susceptible control, Coker 312_WI; susceptible infested with viruliferous whitefly. Error bars represent the SD of three independent experiments.

We used RNA-seq experimental data from Mac7 (*G. hirsutum* accession, resistant to CLCuD)^50^ and NIAB-Karishma (a mutant Coker 312 *G. hirsutum,* highly susceptible to CLCuD)^49^ for expression analysis (Fig. S12). The RNA-seq data include viruliferous whitefly infestation for cotton leaf curl virus disease (CLCuD), Pakistan's threat to cotton production. The expression profiling and comparison demonstrated that 86 CRK genes and 32 genes are expressed in Mac7 and NIAB-Karishma, respectively. The comparative study revealed that most CRK genes showed increased expression in resistant (Mac7) than susceptible NIAB-Karishma. For instance, *GhCRK026, GhCRK013, GhCRK007, GhCRK116, GhCRK108, GhCRK099, GhCRK082, GhCRK072,* and *GhCRK096* were differentially upregulated in resistance under CLCuD disease treatment (Fig. S13, Table S33). The quantitative real-time expression analysis of selected genes displayed intriguing findings. Of the nine selected genes (Table S34), only three genes (*GhCRK093, GhCRK82,* and *GhCRK096*) showed some level of expression in Coker 312 (susceptible accession) and their transcripts level decreased when treated with CLCuD. However, all 9 genes showed increased expression in Mac7 (resistant accession) in control as well as infested sample, and some genes (*GhCRK059, GhCRK081,* and *GhCRK087*) were upregulated under CLCuD (Fig. 8). Generally, we concluded that the CRK genes in Mac7 and Coker are shown to be involved in CLCuD stress.

## Discussion

Cotton (*Gossypium* sp.) is a worldwide economical crop that produces raw fiber and seed oil for the textile and oil industries. But the environmental stresses including biotic (insect, pest, virus, and bacteria) and abiotic (drought, salinity, heat, and cold) are limiting its growth and yield. Thus, the improvement of resistant genetic makeup is essential for high-quality cotton production. CLCuD is one of the major biotic stresses in Asia such as Pakistan and India and this deadly virus decreases cotton yield several-fold every year. Therefore, identification, characterization, and functional analysis of stress-responsive genes are the top targets of cotton researchers. Several genome-wide association studies have been conducted to find important genes involved in different agronomical traits such as fiber yield and improvement, gossypol content, drought, and salt stress-resistant. Similarly, many important resistant QTLs and markers against different abiotic and biotic stresses have been discovered^63^. However, very little is known about the CLCuD resistant mechanism. The current study also provided important data for further functional analysis against CLCuD.

The biotic stress signal in plants observed by pattern recognition receptors (PRRs) also plays a vital role in activating plants' immunity. PAMPs are the biotic stress signals that activate a combination of immune receptors complexes and plant immune response signaling pathways^64^. The pathways that respond to early immune signaling are kinase and transcriptional gene regulation^65^. Antifungal proteins are also called *CRKs* (Cysteine (C)-rich receptor-like kinases gene) and *DUF26* or *Gnk-2*. *CRKs* are an important class of receptor-like kinase (*RLKs*) that play vital roles in disease resistance in plants. Despite the known role of *CRKs* in plant resistance^17,21,22,66–68^, there is a big gap in a genome-wide comparative study of *CRKs* in *Gossypium* species, which was covered in this study. The genomic analysis effectively transfers knowledge from one taxon to another, allowing for a faster pace of gene discoveries associated with disease resistance.

The current study identified *CRKs* in five *Gossypium* sp. and classified them according to domains architectures. A total of 437 CRK genes have stress-antifungal domains in *G. arboreum* (60 genes)*, G. raimondii* (74 genes)*, G. herbaceum* (65 genes)*, G. hirsutum* (118 genes), *and G. barbadense* (120 genes). A similar study of CRK was also conducted by Ting et al. and Hussain et al.^37,69^, but several gaps were filled by this study as we identified an increased number of CRK genes compared to the previous studies. In addition, several additional bioinformatics and expression analyses were also added in the current work. The number of *CRKs* in tetraploids was not more than two-fold of diploid species that may be due to gain and loss of the gene during polyploidization of two diploid genomes (A and D genome) to make the tetraploid genomes (AA_t_DD_t_)^70^. Furthermore, the segmented and tandem duplications influence the development of multiple *CRKs* gene families, including the *RLKs* family^71^. The gene structure analysis identified exon, intron, and UTR regions of the genes. The length of intron and exon is related to the phylogenetic tree's construction, and it is particularly high in *G. raimondii*^72^*.* The comparative analysis showed both differences and similarities in the exon number that might be related to their function and conservation^73^. Chromosomal mapping of *CRKs* showed their abundance on a few chromosomes like Chr6, Chr10, and Chr11 in all five species. Similar chromosomal mapping was also reported by Ting et al.^69^. The region behind the higher density of genes on some species might be due to segmental duplications that occurred between non-allelic chromosomes in cotton. Furthermore, the DUF26 containing genes normally show tandem duplication^74^.

In this study, we have classified *CRKs* in all five species in three different ways, conserved domains absence and presence (DAP), conserved domain repeats (DR), 3rd classification based on Aleksia et al.^15^. Based on the first and second classifications, we have identified several *DUF26* associated decoy domains, like *ALMT* (aluminum-activated malate transporter domain)^75^*, FusC_2 (*Fusaric acid resistance protein-like domain*)*^76^*, Cript* (Microtubule-associated protein domain)*, FYVE* (evolutionarily conserved double-zinc-binding domain) which are functionally well-characterized protein domains and are involved in diver biological function including efflux of organic acids^77^, salt stress tolerance and the regulation of malic acid content^78^, linkage of mRNA transporter to endosome trafficking^79^. Such resistance decoy domains also reported in *Gossypium*^37^ may provide additional features to CRK genes for plants adaptation. Molecular phylogenetic analysis and OrthoFinder results suggested a significantly divergent evolutionary history of CRK genes in five species. The species-based phylogenetic tree of *CRKs* suggested *G. arboreum* as the ancestor of other *Gossypium* sp. and the othogroups also found some species-specific groups. However, the five species showed a close relationship in sequence similarity, possibly due to the origination of *CRKs* from common ancestors. The evolution of genes is mediated by sequence exchange, tandem or segmental duplication events, or gene conversion^80–82^.

A single-nucleotide polymorphism (SNPs) is the simplest form of genetic variation among individuals that can prompt minor changes in phenotypic, physiological, and biochemical characteristics. These mutations in the gene sequence alter the amino acid sequence, which may change the function of the gene. Several SNPs were identified and used as a genetic marker for the identification of qualitative trait loci (QTLs) associated with multiple agronomic features of cotton including fiber quality and quantity, resistance to biotic and abiotic stresses^83–87^. However, very little is known about SNPs associated with biotic stress resistance. So, we also have found several SNPs in CRK genes of resistant accession of *G. hirsutum* in comparison with the Coker 312 (highly susceptible) and TM-1 reference genome. We suggest that these SNPs may have a significant role in plant adaptation under CLCuD. However, further experimental validation is required to confirm their role as selection markers.

miRNA contains 17 to 24 nucleotides (nt) and is an important gene regulatory factor in plants^88^. miRNAs take part in diverse biological functions of plants at different transcriptional and translational levels^88–91^. They also play essential roles in developing immunity against pathogens succeeding the endogenous defense-related genes and down-regulating the pathogens of the exogenous viral plants^92–94^. The miRNA target sites in *CRKs* provide essential primary data for understanding *CRKs* regulation under various stresses. In cotton *CRKs*, we found different miRNA families in diverse cotton species such as *miR172*, *miR1373*, *miR169*, and *miR164* in *GaCRKs*, whereas *miR172, miR159, miR169,* and *miR397* in *GhCRKs*. The genotype-depended response of miRNA to biotic and abiotic stresses varies from cultivar to cultivar^95^. Another study of miRNA microarray in cotton found high expression of *miR156, miR169, miR535,* and *miR827* under salinity treatment^96^. The *miR172b-SSR* was used as a biomarker for identifying the different responses of rice cultivars under salt stress^97^. In agreement with the literature, several miRNA families were identified which could target CLCuDV genes with perfect and near-perfect complementarity^98^. In addition, the *miR172* initiates floral growth and modifies reproductive growth and the co-regulation of *miR*156 and *miR172* in the origination and improvement of cotton plants^99^. So, the identification of miRNAs in cotton *CRKs* would be helpful for understanding the post-transcriptional regulation of *CRKs* under diverse stress conditions. We also summarize based on the miRNA target site prediction and expression profiling of miRNA, there might a strong correlation between miRNA expression and functional regulation of cotton CRK genes. As we have observed that *CRKs* have the highest target side for the *miR172* family and in the meanwhile the *miR172* family has high expression in most of the tissue, demonstrating its role in the regulation of various biological mechanisms.

The difference in the number of genes and classes among 35 plants species is due to the expansion of CRK families through small-scale duplication, genome fractionation, and genetic drift which cause due to whole genome multiplications^16^. One prediction suggests that the duplicated genes under dosage balance exhibit fewer expressions than other duplicates and *RLKs* can function not only in defense but also in development and abiotic stress responses^100,101^. Hence, to determine the functional conservation and putative role of *CRKs* in cotton development and adoption under stresses, publicly available expression data of tissues and stress treatments were used and highlighted several tissue-specific and stress-specific expressions of *CRKs* in cotton. For instance, a cluster of *CRKs*; *GhCRK053, GhCRK083, GhCRK094, GhCRK038, GhCRK110, GhCRK039, GhCRK041, GhCRK068, GhCRK093,* and *GhCRK013*, induced transcriptionally under salt, drought, heat, and cold stresses in *G. hirsutum*. Previous studies reported that *AtCRKs* are transcriptionally induced under abiotic stresses such as salt, drought, UV light, heat, salicylic acid^17–21^. In agreement with such previous studies of *CRKs* in Arabidopsis, we also found important several stress-responsive cotton *CRKs*. In addition to abiotic stresses, we also reported the important cotton *CRKs* response under biotic stresses including cotton leaf curl disease for instance the *G. hirsutum CRKs*; *GhCRK026, GhCRK013, GhCRK007, GhCRK116, GhCRK108, GhCRK099, GhCRK082,* and *GhCRK072* differentially upregulated in resistant accession while down-regulated in susceptible accession under CLCuD disease treatment. The genetic variants analysis also identified several SNPs and InDels in these putative genes. Furthermore, the quantitative real-time expression analysis also validated the RNA-seq analysis of *GhCRKs*. All selected *GhCRKs* were transcriptionally upregulated in resistant accession while either down-regulated or did not show any transcript in the susceptible accession. In literature, it is reported that a subset of *CRKs* is strongly induced in response to pathogens and PAMPs treatments^19,20^ and overexpression of *AtCRK4, AtCRK5, AtCRK6, AtCRK13*, and *AtCRK36* showed enhanced resistance to the bacterial pathogen *Pseudomonas syringae* as well as also activated the early and late PTI responses^17,21,22^. Henceforth, the comparative expression of *CRKs* in resistant and highly susceptible cotton provided important *CRKs* candidates. The coordination of these *CRKs* during plant immune response suggested that they cooperate in plant defense signaling. Furthermore, the molecular docking of *CRKs* with CLCuD viral proteins also demonstrated their direct interaction. As CRK proteins possess extracellular domains, which are involved in protein–protein interaction and signal perceptions^12^, the transmembrane domains and intracellular domains transduce and activate MAPK pathways for activation of the plant. The host–pathogen interaction and expression data showed coordination of these putative genes in plant immune signaling. Thus, the *GhCRK057, GhCRK059, GhCRK058, GhCRK081, GhCRK008,* and *GhCKR087* might be a potential marker for CLCuD resistant genotype. The current study provided a deep insight into *CRKs* in *Gossypium* sp. The different ploidy level of *Gossypium* species has different resistance level, for instance, the diploid species like *G. arboreum*, *G. raimondii*, and *G. herbaceum* are naturally resistant to several biotic and abiotic stresses while the tetraploid cotton-like *G. hirsutum* is susceptible to multiple stresses and the *G. barbadense* is highly susceptible to environmental stresses. Thus, the comparative study of stress-responsive genes *CRKs* in cotton is essential for improving cotton growth and development.

## Conclusion

The current study identified a total of 437 Cysteine-rich receptor-like kinases (*CRKs*) encoding genes in five *Gossypium* sp. The structural and domain-based classification identified several novel domain architectures in *Gossypium* sp*.* The genome mapping and genetic diversity (SNPs and InDels) provided important data for cotton breeders and the expression profiling under different environmental stresses and their validation through qPCR under CLCuD demonstrated a putative role in cotton growth and development. The miRNA target site prediction will help to understand the regulation of *CRKs* in specific tissues. We have provided detailed computational and experimental studies on *CRKs* in the five species; however, further individual gene functional analysis is required to understand the *CRKs* mechanism in cotton plants' adaptation.

## Supplementary Information

Supplementary Figures.

Supplementary Tables.

## Data Availability

Source data for all the graphs included in this paper are available as Supplementary Data in Excel format. All other data are available from the corresponding author upon reasonable request. It is also stated that there are no ethical issues that required permissions or licenses to complete this work.
