# Supplementary material for: Genome wide study of cysteine rich receptor like proteins in Gossypium sp
Source: Sci Rep. 2022 Mar 22;12:4885. doi: 10.1038/s41598-022-08943-1 (PMC8941122; doi:10.1038/s41598-022-08943-1)
Supplement: Supplementary file 1 — Supplementary Figures. [file 41598_2022_8943_MOESM1_ESM.docx]

**Supplementary Data**

**Genome wide study of cysteine rich receptor like proteins in *Gossypium* sp.**

Athar Hussain^1,2^*, Naila Asif^3^, Abdul Rafay Pirzada^3^, Azka Noureen^2,4^, Javeria Shaukat^3^, Akif Burhan^3^, Madiha Zaynab^5^, Ejaz Ali^6^, Koukab Imran^3^, Ayesha Amin^7^, Muhammad Arslan Mahmood^2^, Aquib Nazar^3^, M. Shahid Mukhtar^8^

^1^Genomics Lab, School of Food and Agricultural Sciences (SFAS), University of Management and Technology (UMT), Lahore, 54000, Pakistan

^2^National Institute for Biotechnology and Genetic Engineering (NIBGE), College of Pakistan Institute of Engineering and Applied Sciences (PIEAS), Faisalabad, 38000, Pakistan

^3^Department of Life Sciences, School of Science, University of Management and Technology (UMT), Lahore, 54000, Pakistan

^4^PMAS-Arid Agriculture University Rawalpindi, Rawalpindi, 46300, Pakistan

^5^Shenzhen Key Laboratory of Marine Bioresource & Eco-Environmental Sciences, College of Life Sciences and Oceanography, Shenzhen University, Shenzhen 51807, China

^6^Center of Excellence in Molecular Biology, University of Punjab, 54000, Lahore, Pakistan

^7^Office of Research Innovation and Commercialization, University of Management and Technology (UMT), Lahore, 54000, Pakistan

^8^Department of Biology, the University of Alabama at Birmingham, 1300 University Blvd., Birmingham, AL, 35294, USA


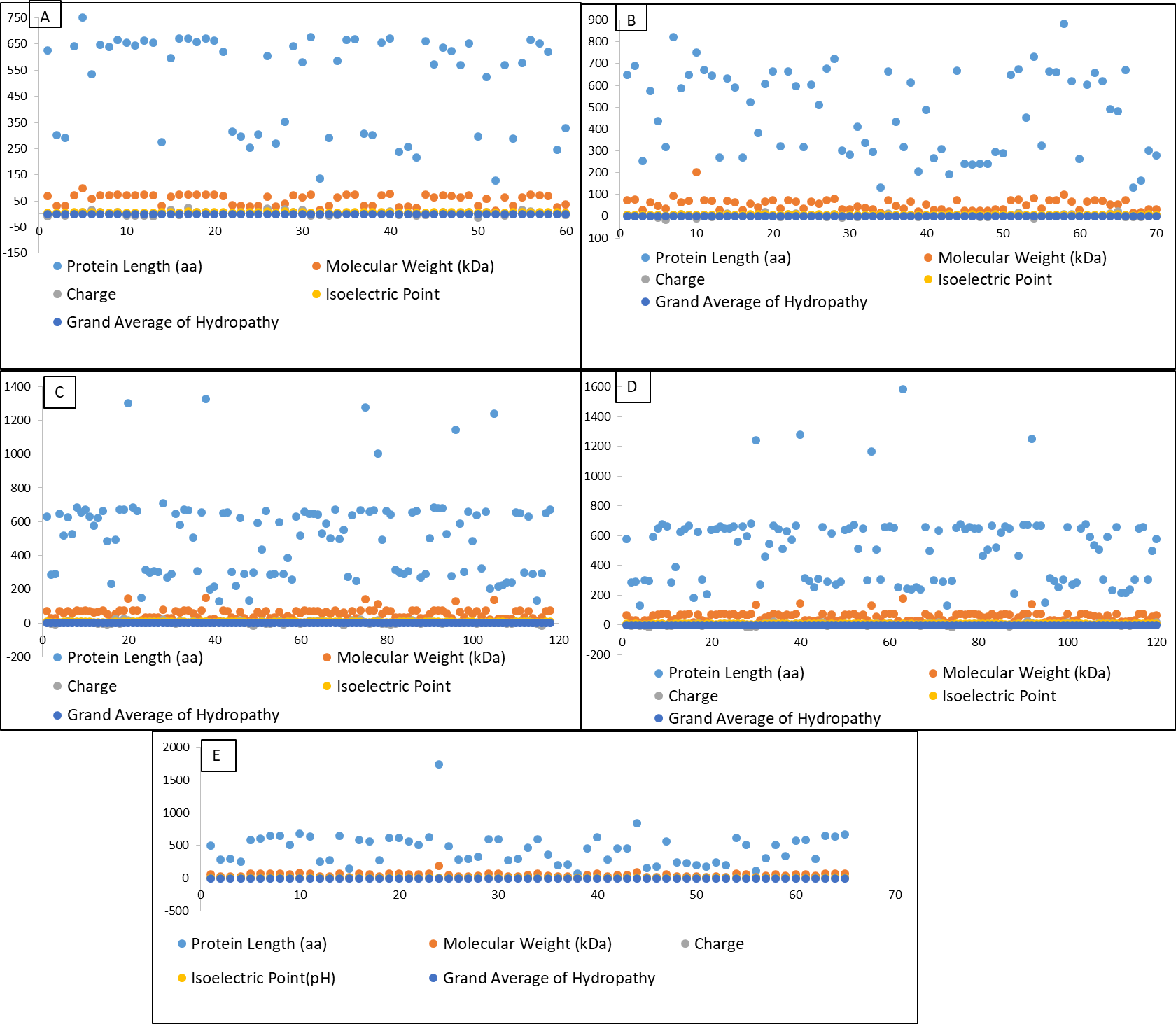


**Figure S1** Protein statistical values ranges of antifungal protein in (A) *G*. *arboreum*, (B) *G. raimondii*, (C) *G. hirsutum*, (D) *G.* *barbadense,* and (E) *G. herbaceum*


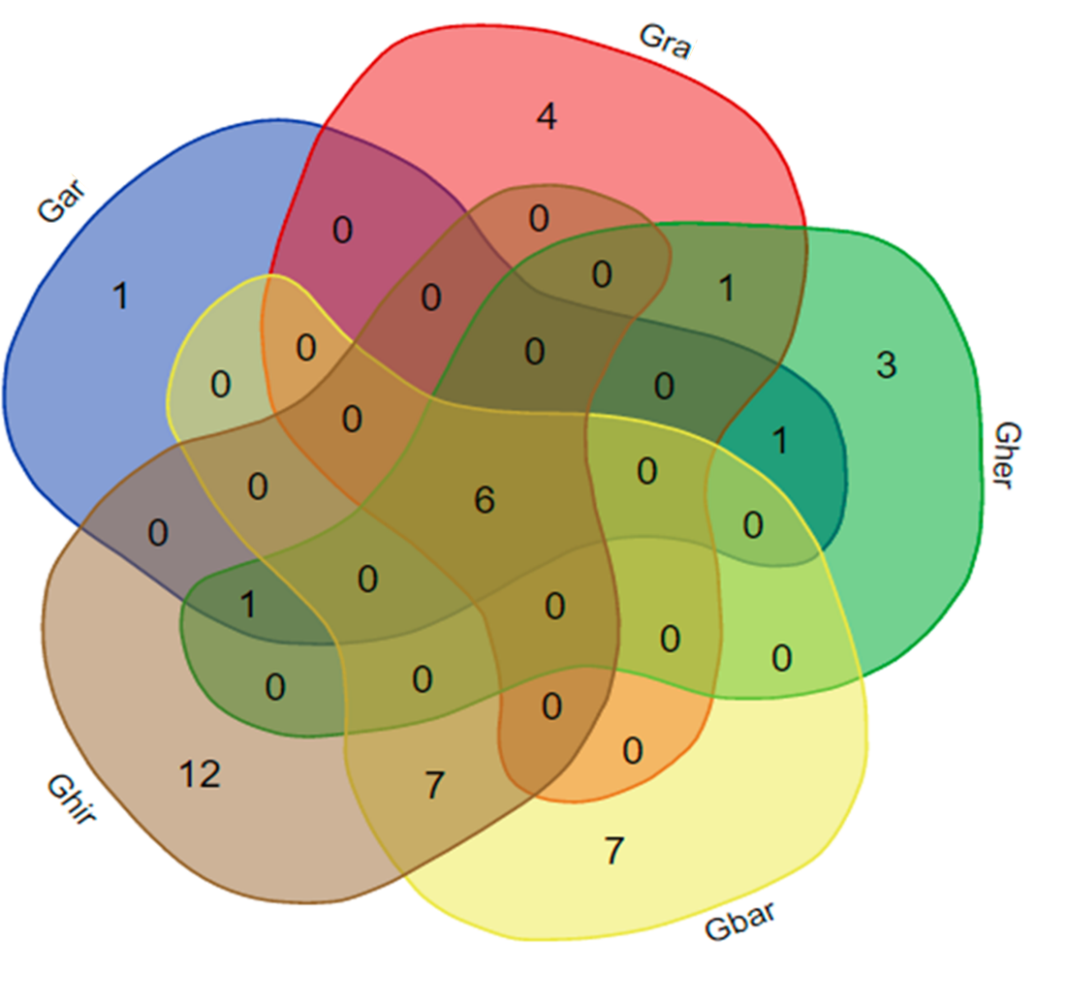


**Figure S2** Common and species-specific domain architectures of *CKRs* in species including *G. arboreum* (*Gar*), *G. raimondii* (*Gra*), *G. hirsutum* (*Ghir*), *G. barbadense* (*Gbar*) and *G. herbaceum* (*Gher*).


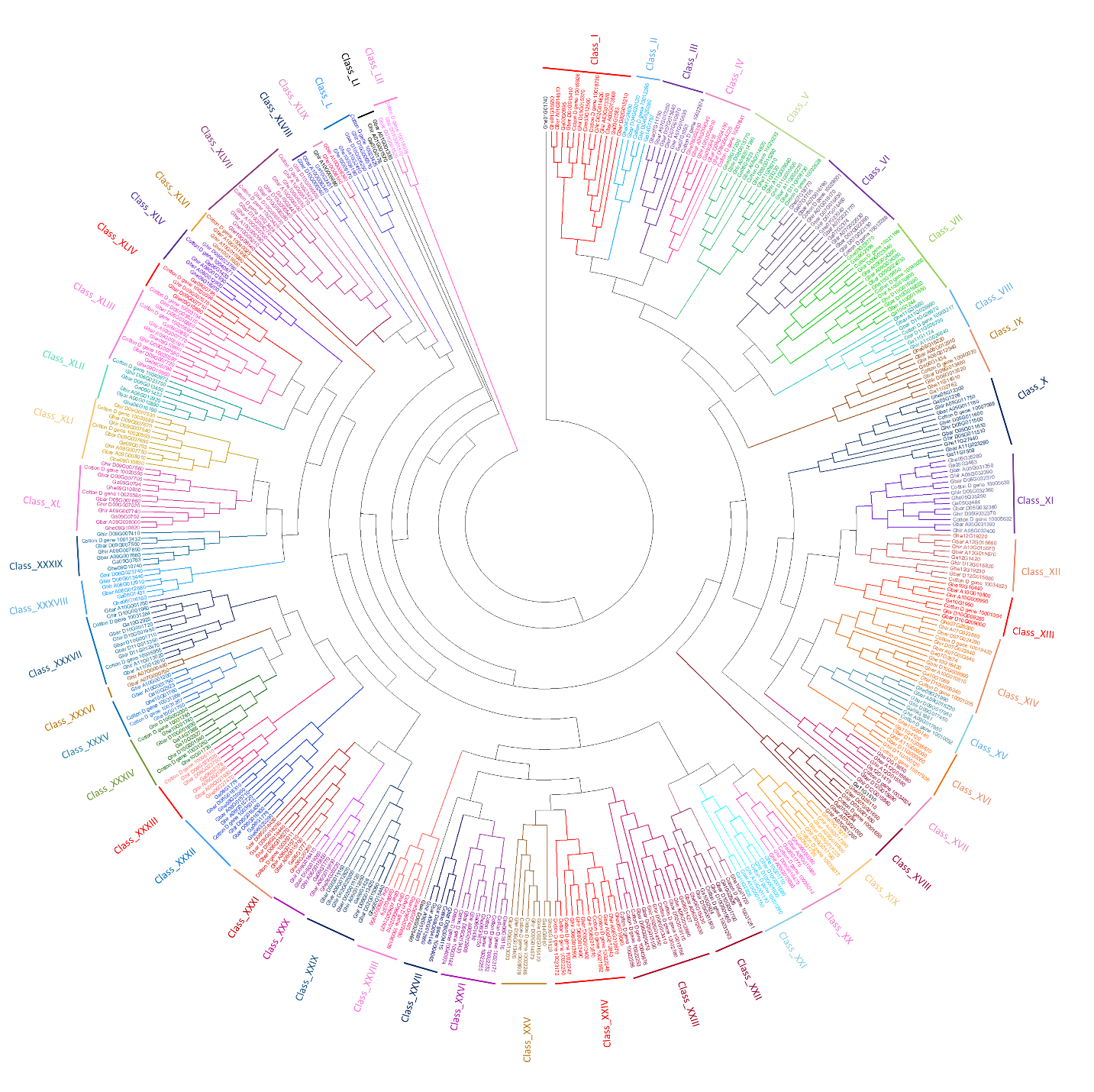


**Figure S3** Accumulative phylogenetic tree of cotton CRKs. Different colors are representing subclasses of cotton CRKs


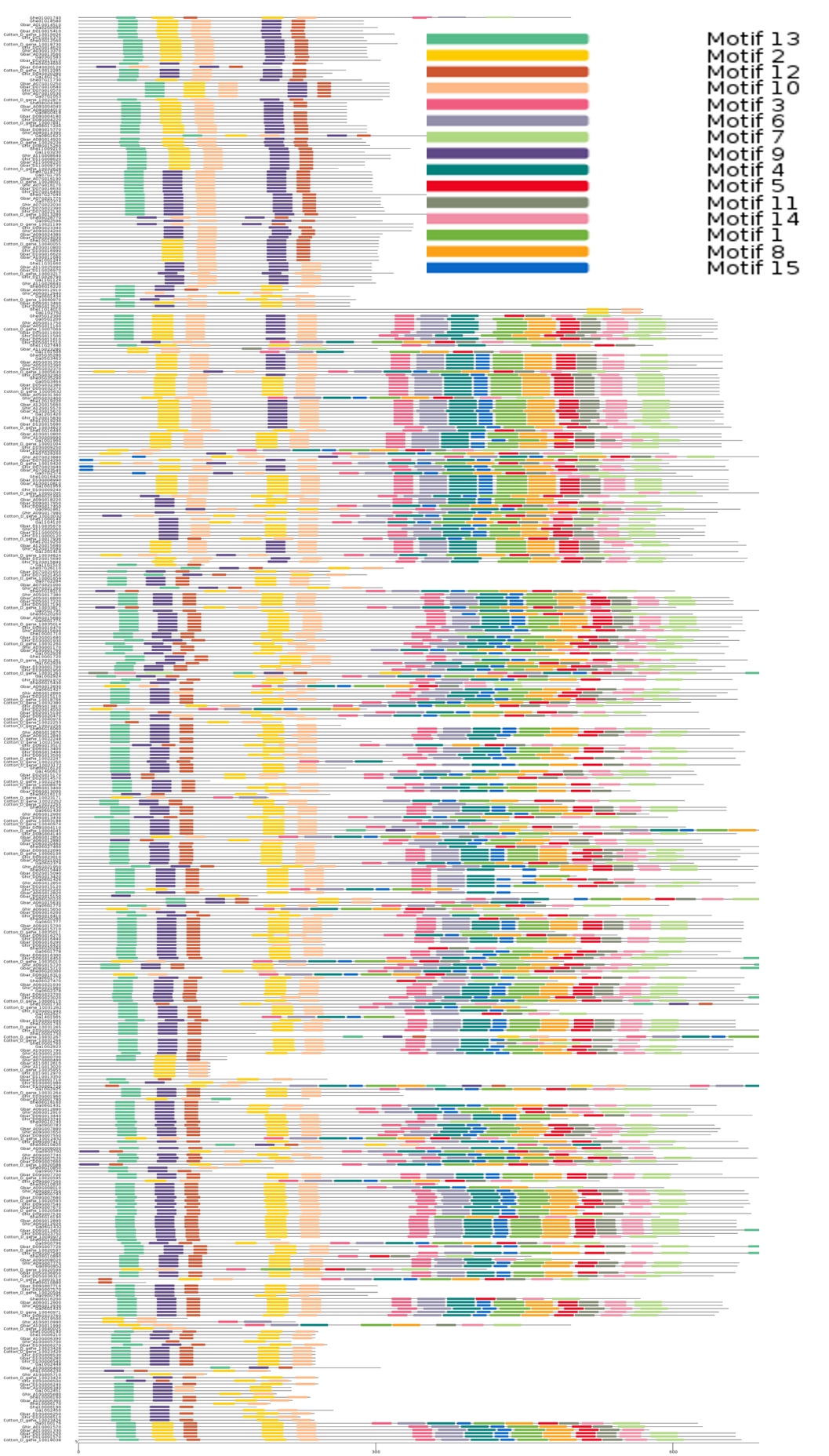


**Figure S4** De-novo conserved MEME motifs, found in cotton CRKs proteins.


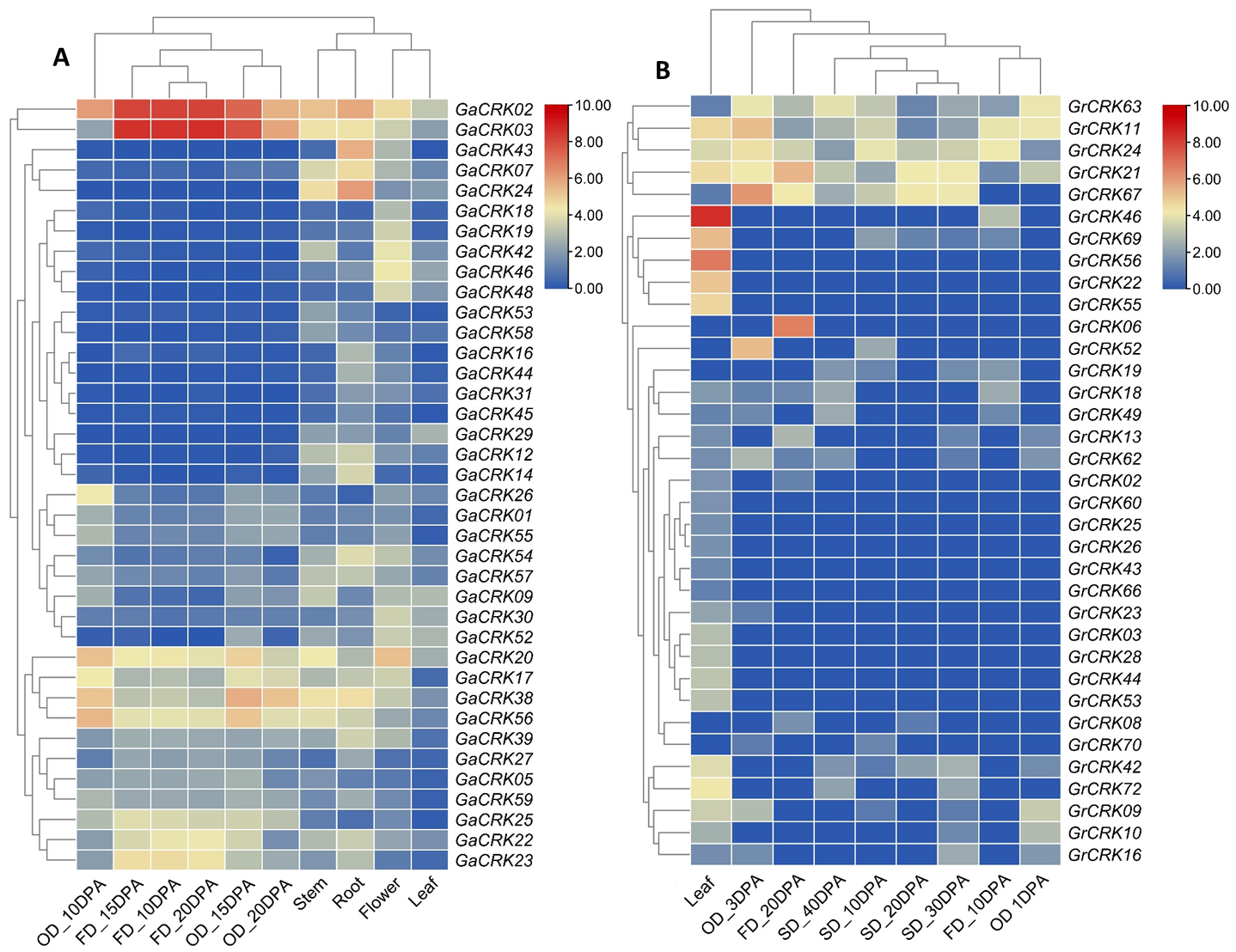


**Figure S5** Tissue-specific expression profiling of CRKs. (**A**) *G. arboreum,* (**B**) *G. raimondii.* OD; ovule development, FD; fiber development, SD; seed development DPA; day post anthesis


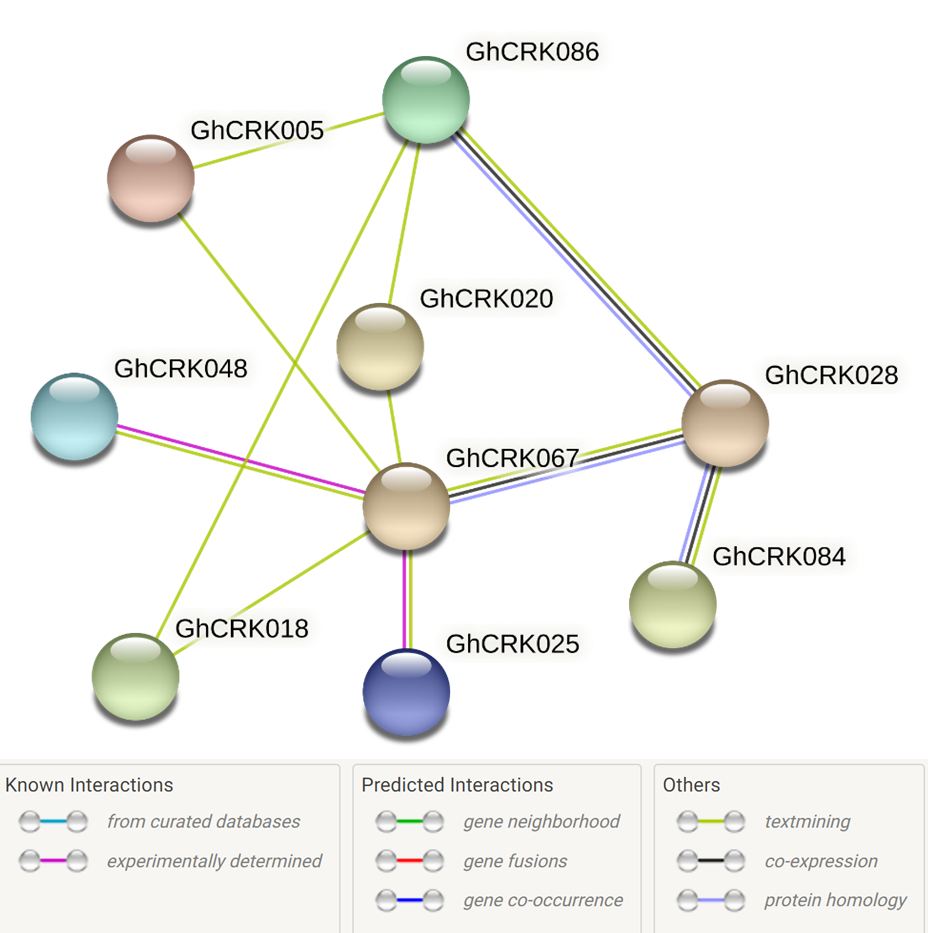


**Figure S6** Protein-protein-interaction network of GhCRKs


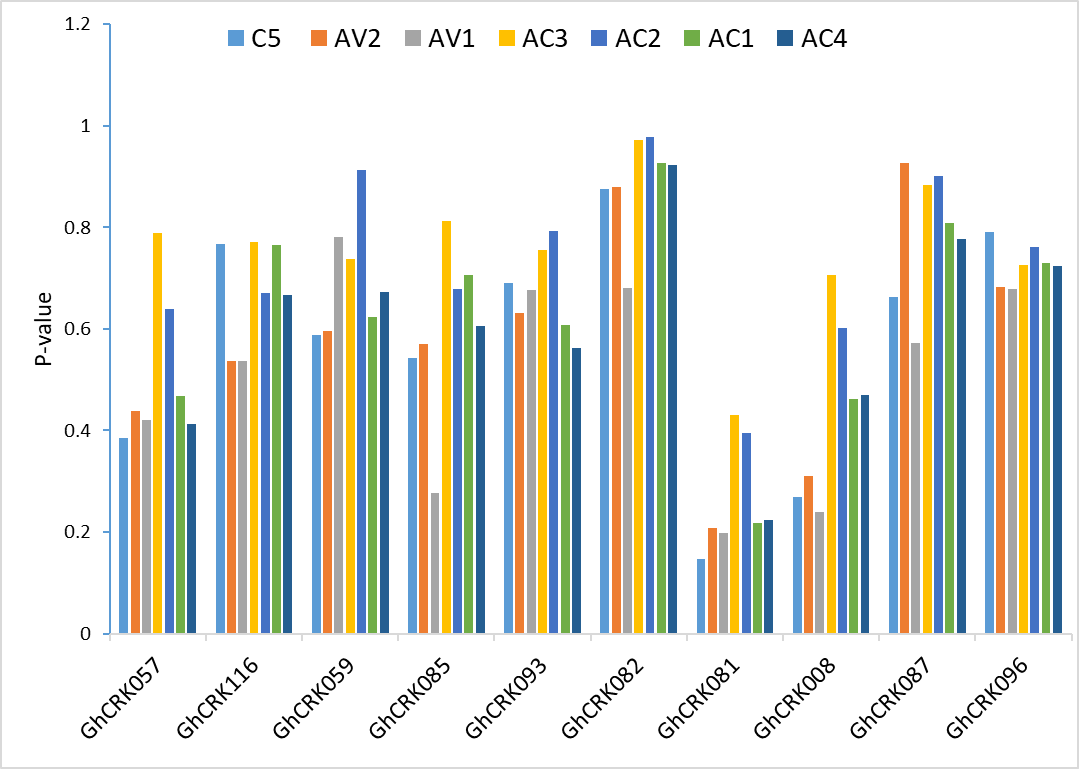


**Figure S7** Protein-protein interaction between host GhCRK proteins and CLCuD viral proteins.


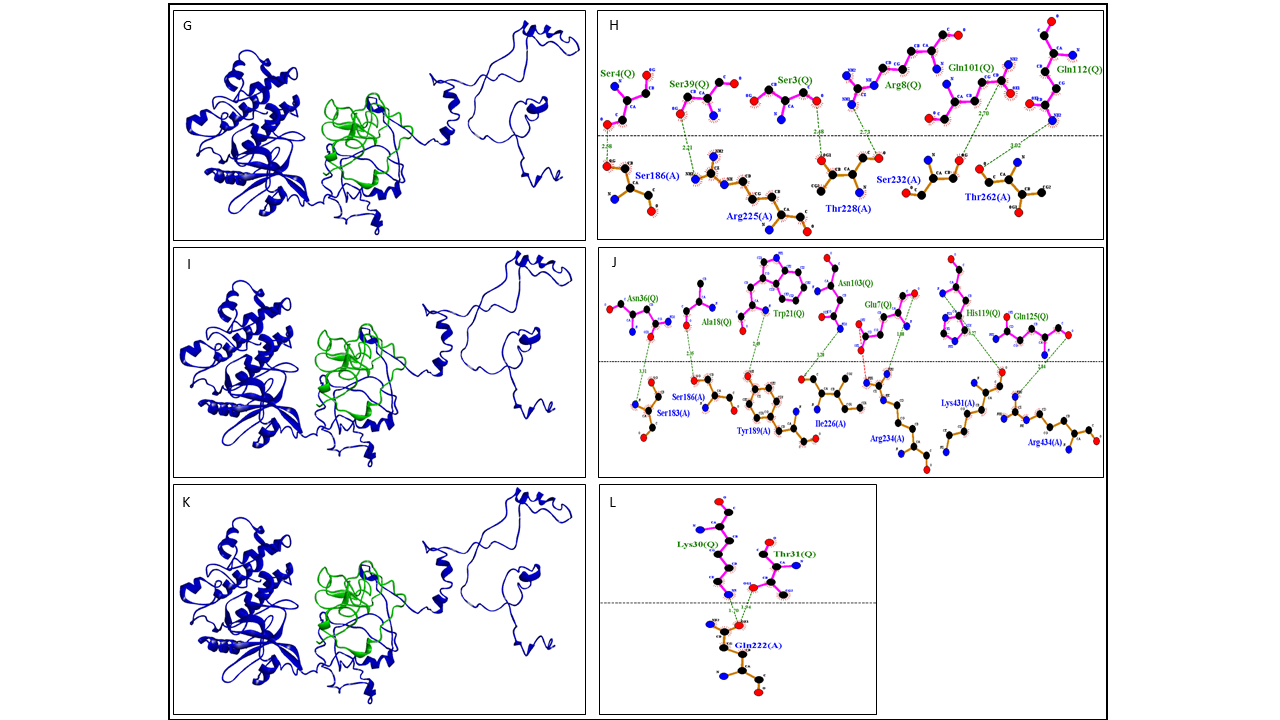


**Figure S8** Host (Blue)-Pathogen (Green) interaction (G) CRK102 (Green) -AC2 (Blue) Complex. (H) CRK102 (A)- AC2 (Q) 2D interaction graph (I) CRK102 (Green)-AC3 (Blue) Complex. (J) CRK102 (A)-AC3 (Q) 2D interaction graph. (K) CRK102 (Green)-AV2 (Blue) Complex. (L) CRK102 (A) – AV2 (Q) 2D interaction graph.


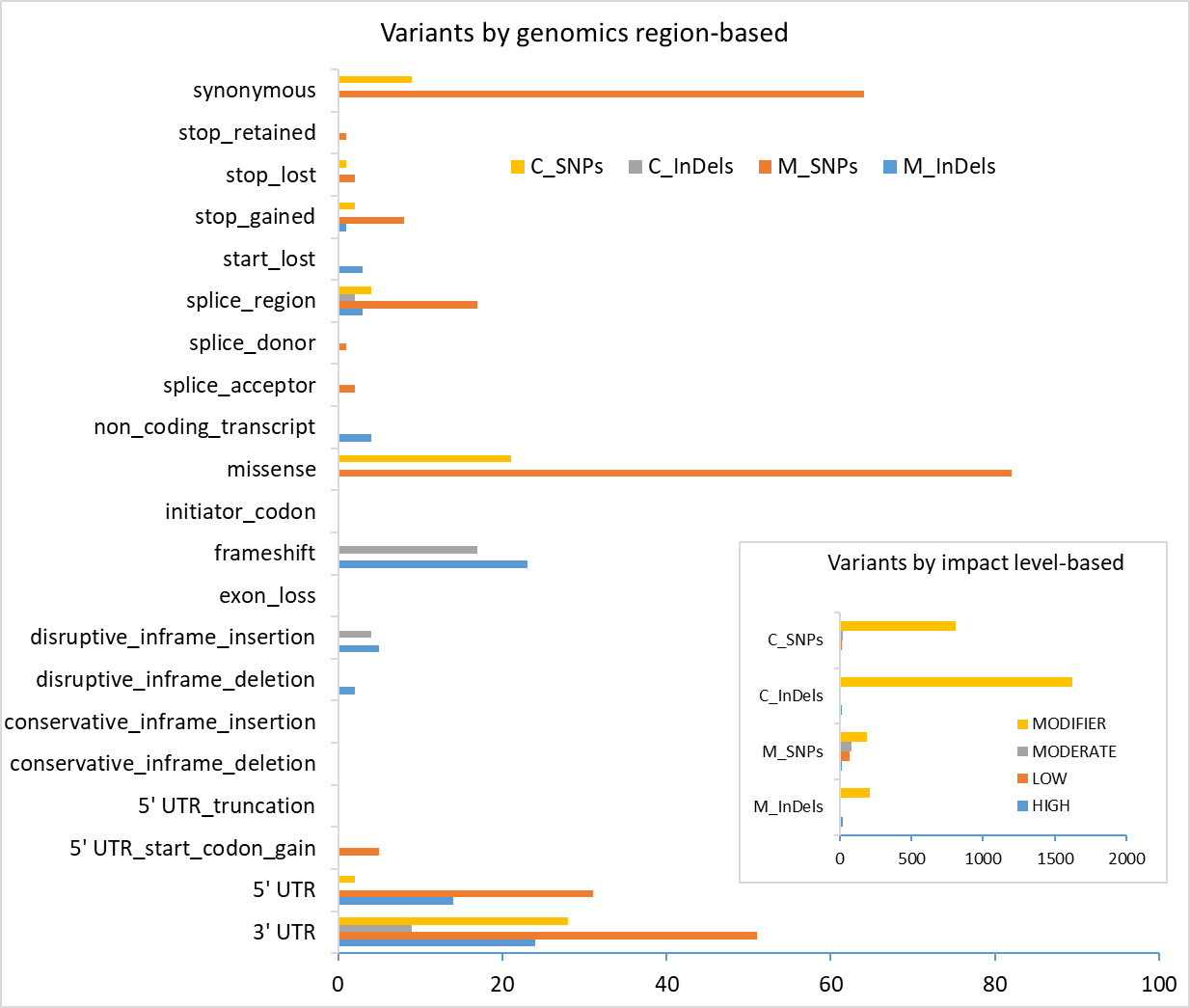


**Figure S9** Genetic variants of CRK genes found in Mac7 (resistant to CLCuD) and Coker 312 (highly susceptible to CLCuD) accessions. C; Coker 312, M; Mac7.


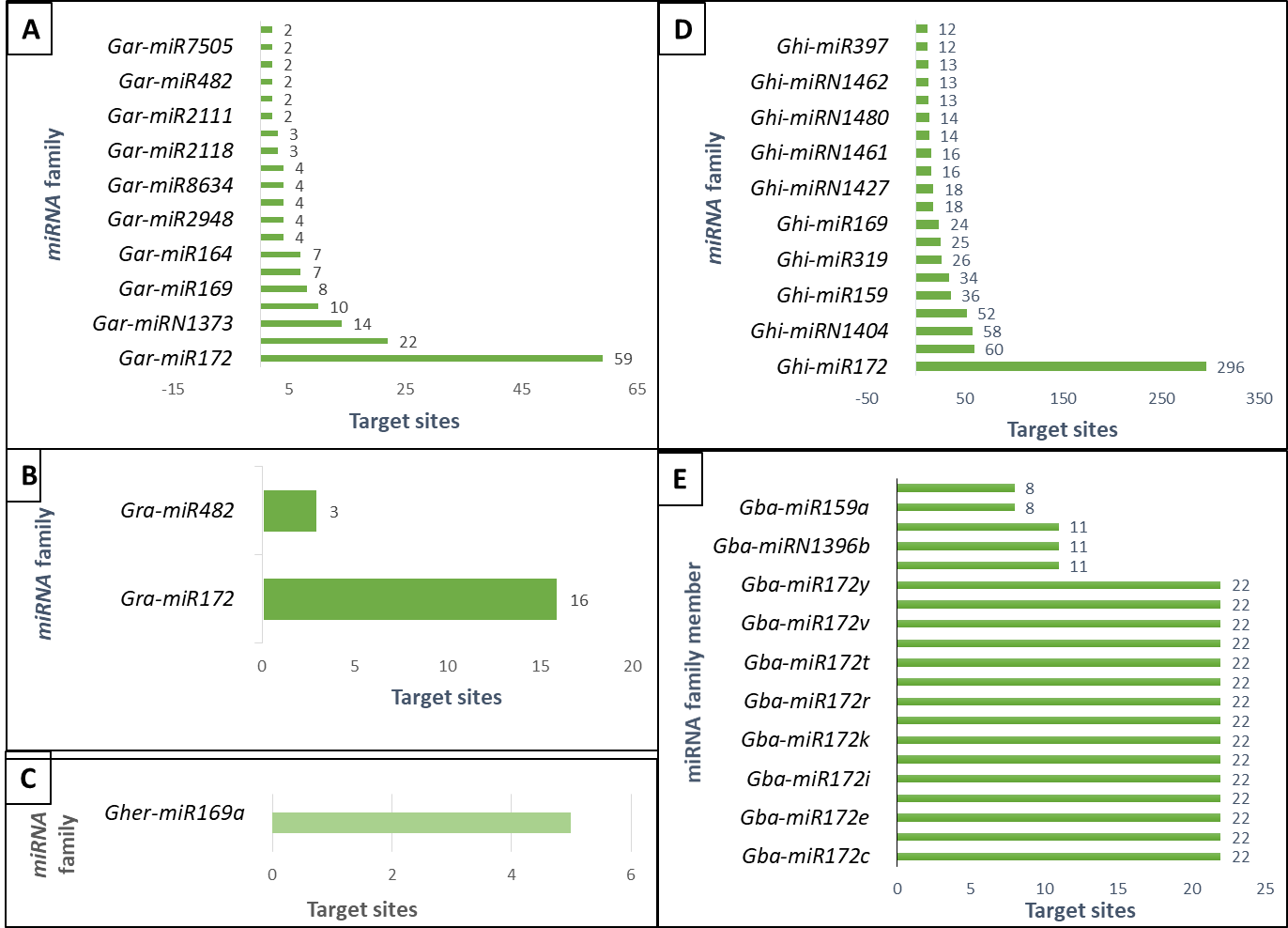


**Figure S10** Micro RNA families and their number of targets in CRKs coding sequence. (A) *G. arboreum*, (B) *G. raimondii*, (C) *G. herbaceum,* (D) *G. hirsutum* and (E) *G. barbadense*

**
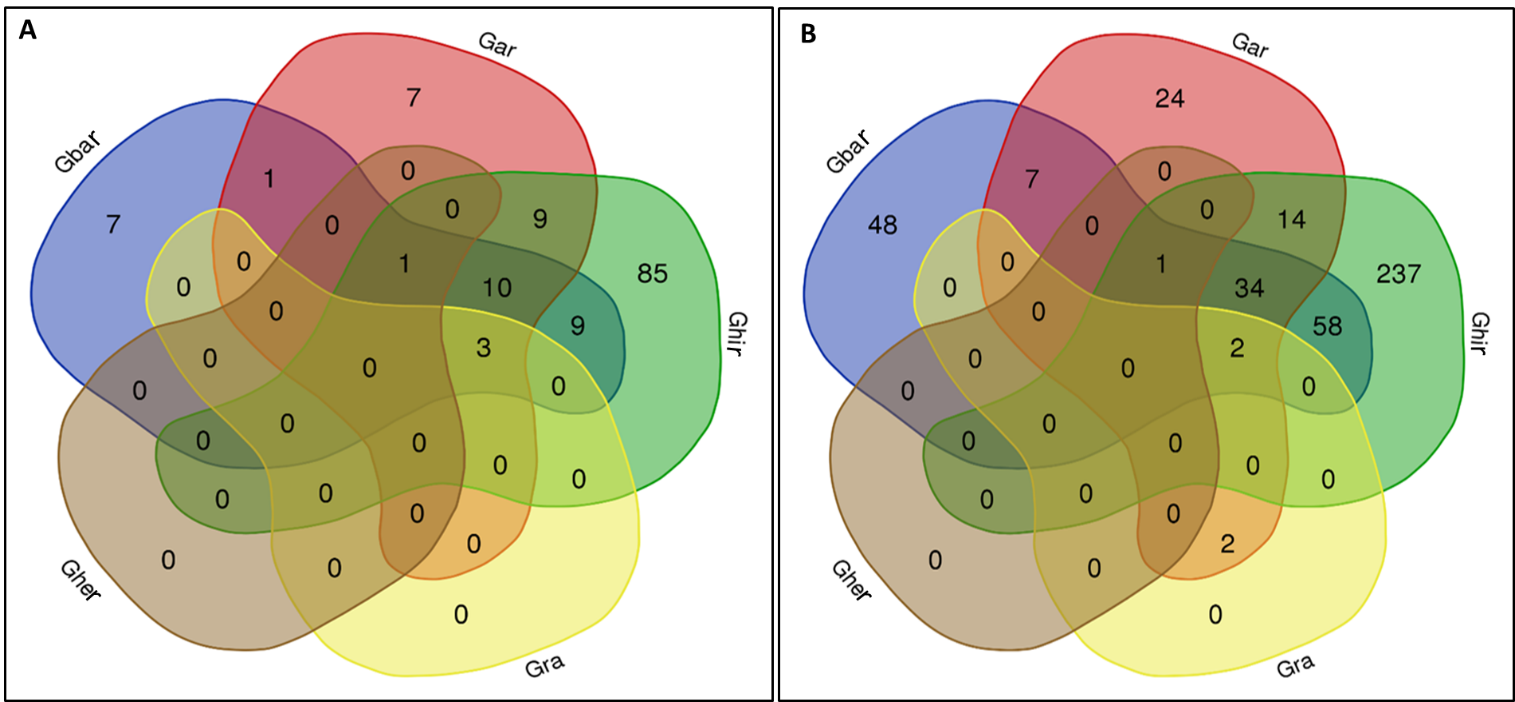
**

**Figure S11** Identified micro RNA comparison among five species. (A) miRNA families (B) miRNA family members.


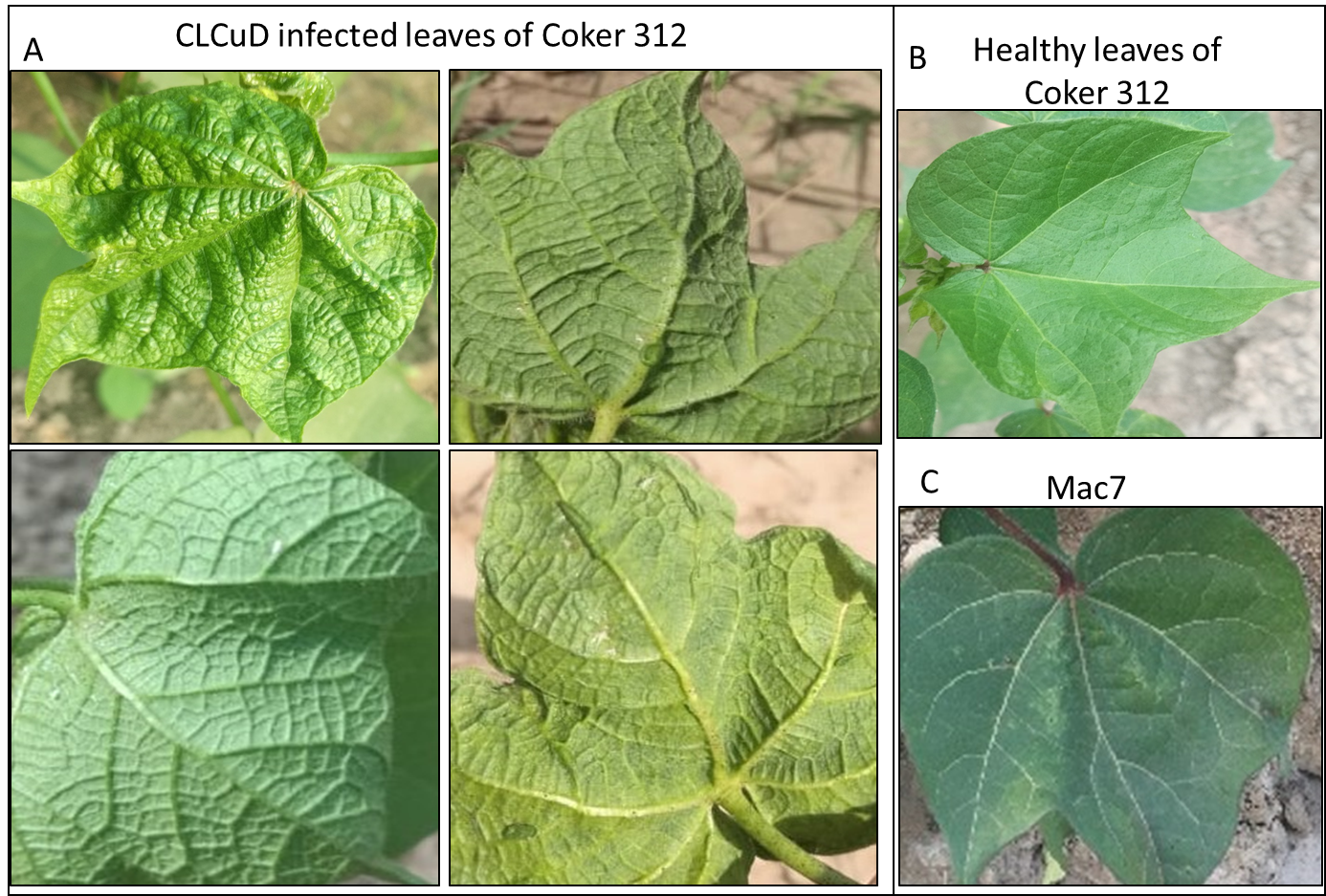


**Figure S12** Cotton leaf of susceptible and resistant G. hirsutum accessions under cotton leaf curl disease attack. (A) Infected leaf of Coker 312 (susceptible to CLCuD) (B) Healthy leaf of Coker 312 (C) Mac7 leaf under CLCuD (resistant to CLCuD). These are photos of cotton plants leaf grown in NIBGE net house and photos are taken by Mr. Hussain.


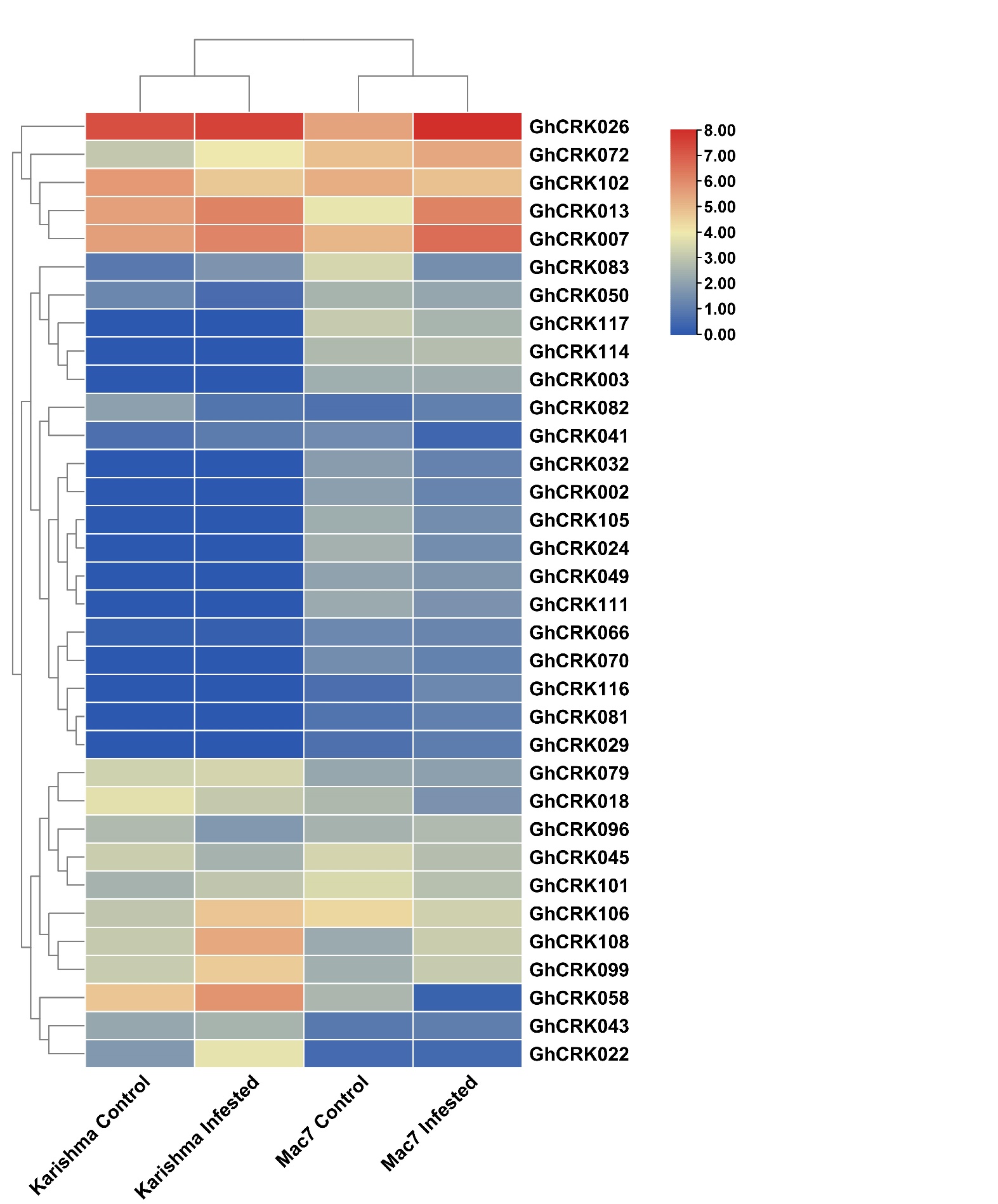


**Figure S13** Expression profiling of *G. hirsutum CRKs* under cotton leaf curl disease infestation. Mac7; a CLCuD resistant accession, Karishma; a highly susceptible accession. Values are given in FPKM with log2 base.
